# Low‐Frequency Microwave Absorption Composites

**DOI:** 10.1002/advs.202511580

**Published:** 2025-07-23

**Authors:** Mukun He, Kaiyu Zhang, Hua Qiu, Hua Guo, Xiangcheng Li, Yongqiang Guo, Yali Zhang, Junwei Gu

**Affiliations:** ^1^ Shaanxi Key Laboratory of Macromolecular Science and Technology School of Chemistry and Chemical Engineering Northwestern Polytechnical University Xi'an Shaanxi 710072 P. R. China; ^2^ Queen Mary University of London Engineering School Northwestern Polytechnical University Xi'an Shaanxi 710072 P. R. China; ^3^ Key Laboratory of High Temperature Electromagnetic Materials and Structure of MOE Wuhan University of Science and Technology Wuhan Hubei 430081 P. R. China

**Keywords:** composites, L, S, and C bands, low‐frequency microwave absorption, multiple losses

## Abstract

Electromagnetic wave devices and components operating in the low‐frequency band of 1–8 GHz (L, S, and C bands) are widely applied in wireless data communication systems, satellite radar, and other fields. However, traditional low‐frequency microwave absorption materials (MAMs) suffer from issues such as large thickness, heavy weight, difficult impedance matching, insufficient low‐frequency microwave loss, and poor environmental stability, severely limiting use and development. In recent years, researchers construct composite systems combining various types of loss, coupled with component adjustments and structural design strategies, significantly improving the impedance matching and multiple loss synergy effects for low‐frequency MAMs. Based on the above, this review briefly introduces the basic principle of microwave absorption and the influence factors of low‐frequency microwave absorption performance, and systematically reviews the recent research progress of carbon‐based composites, magnetics‐based composites, polymer‐based composites, ceramic‐based composites, and multiphase hybrid composites. Additionally, the intrinsic relationship and mechanisms are discussed in detail between the micro‐structure and macro‐performance of different composites. Furthermore, the key scientific and technical bottleneck problems that need to be addressed in the design and preparation of low‐frequency MAMs are identified. It is expected to provide theoretical guidance for the controlled preparation and performance regulation of low‐frequency microwave absorption composites.

## Graphical abstract

The microwave absorption principles and low‐frequency performance factors are briefly introduced. Recent progress is systematically summarized in five different based composites. Then detailed analysis focuses on relationships and mechanisms about micro‐structure and macro‐performance. Furthermore, the key scientific and technical bottlenecks in designing low‐frequency microwave absorption materials are identified. To provide theoretical guidance for the controlled preparation and performance optimization.

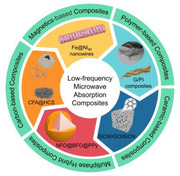

## 1 Introduction

With the advancements of communications, computers, and sensor technologies, microwaves, as a crucial medium for information transmission, are widely used in fields such as communication, medicine, military industry, and microelectronic.^[^ ^1^, ^2^, ^3^, ^4^ ^]^ However, prolonged exposure to high‐power electromagnetic radiation may pose a potential threat to human health and accelerate the performance degradation as well as shortened the service life of electronic devices.^[^ ^5^, ^6^, ^7^ ^]^ In the current wireless communication systems, low‐frequency microwaves (L band: 1–2 GHz, S band: 2–4 GHz and C band: 4–8 GHz) are widely used in WiFi, Bluetooth and 5G communication technologies, and the working frequency bands are mainly concentrated in 2.4–5 GHz.^[^ ^8^, ^9^, ^10^ ^]^ Furthermore, with the continuous development of satellite communication and radar detection technologies, the minimum operating frequency of interstellar radar systems has extended to ≈1.2 GHz.^[^ ^11^, ^12^ ^]^ To effectively reduce the adverse effects of low‐frequency electromagnetic radiation on the human body and equipment, the development of high‐performance low‐frequency MAMs has become an important research direction in the field of microwave protection.

Traditional low‐frequency MAMs face multiple challenges in practical applications. The main limitations include large material thickness, heavy weight, difficult impedance matching, insufficient low‐frequency microwave loss, and poor environmental stability, etc.^[^ ^13^, ^14^ ^]^ Specifically, the excessive thickness and weight are difficult to meet the requirements in aircraft skins, unmanned aerial vehicle stealth design, as well as thinness and lightness in electronic products.^[^ ^15^, ^16^, ^17^ ^]^ Furthermore, high permittivity materials represented by carbon‐based materials and MXene often cause severe impedance mismatch, resulting in incident microwaves being reflected at the material surface rather than absorbed into the material interior, thereby significantly reducing the microwave absorption efficiency.^[^ ^18^, ^19^, ^20^ ^]^ Additionally, traditional magnetic materials (such as iron, cobalt, nickel metals, and carbonyl iron) suffer from a significant decrease in permeability at low‐frequency, making them difficult to fully dissipate microwave energy through magnetic loss, further limiting microwave absorption performance in the low‐frequency band.^[^ ^21^, ^22^, ^23^ ^]^ Meanwhile, the hot and humid environment can intensify the moisture adsorption within the materials, causing the matrix expansion and structural failure, which alters electromagnetic parameters and affects the interface polarization effect.^[^ ^24^ ^]^ These problems seriously restrict the long‐term reliability of traditional low‐frequency MAMs. To address the above challenges, researchers combine different loss‐type MAMs by adjusting the composition of carbon materials, magnetic metals, polymer matrices, ceramic materials, and multiphase hybrid materials. Meanwhile, structural designs such as layered structure, hollow structure, porous structure, and homogeneous structure are introduced to effectively improve impedance matching and achieve the synergistic enhancement of dielectric loss and magnetic loss. In recent years, research on MAMs has developed rapidly, but there is currently a lack of systematic reviews on low‐frequency microwave absorption composites.

**Figure 1:**
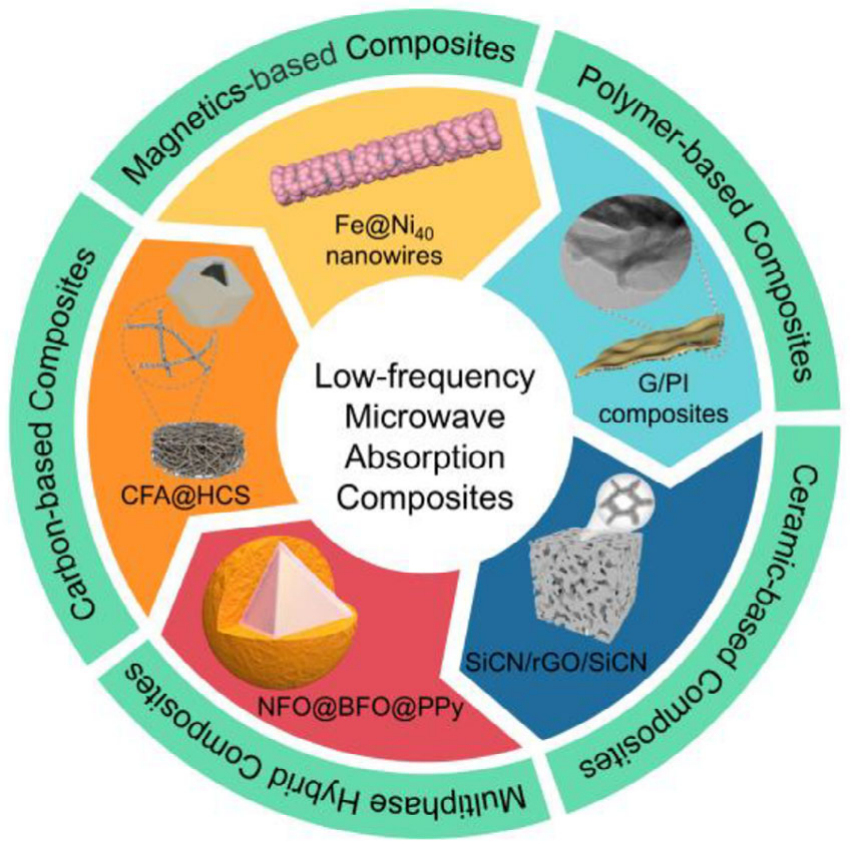
Classification of low‐frequency microwave absorption composites.

Based on the above, this review briefly introduces the basic principle of microwave absorption and the influencing factors of low‐frequency microwave absorption performance, and systematically reviews the research progress of carbon‐based composites, magnetics‐based composites, polymer‐based composites, ceramic‐based composites, and multiphase hybrid composites (**Figure** **1**). The intrinsic relationship of micro‐structure as well as macro‐performance in different composite material systems and the microwave absorption mechanisms are mainly discussed. The key scientific problems and technical bottlenecks that need to be urgently solved in the design and preparation process of low‐frequency MAMs are pointed out, hoping to provide theoretical guidance for the controllable preparation and performance regulation of low‐frequency microwave absorption composites.

## 2 Microwave Absorption Mechanisms and Factors Influencing Low‐Frequency Performance

### 2.1 Fundamental Principles of Microwave Absorption

Three primary phenomena occur when incident microwaves enter MAMs. A portion of microwaves is reflected by the surface of materials (reflected waves), a portion is absorbed inside the materials (absorbed waves), and the remaining portion penetrates the materials to become transmitted waves.^[^ ^25^, ^26^, ^27^ ^]^ The intensity of the absorbed waves depends on the ability to attenuate microwaves for materials, and this attenuation is mainly achieved through dielectric loss and magnetic loss.^[^ ^28^, ^29^, ^30^ ^]^ Dielectric loss is usually determined by conductive loss, polarization relaxation, and multiple scattering.^[^ ^31^, ^32^, ^33^ ^]^ Magnetic loss is typically determined by eddy current loss, natural resonance, and exchange resonance.^[^ ^34^, ^35^, ^36^ ^]^ The synergistic effect of these microscopic loss mechanisms is ultimately reflected through macroscopic performance indicators, including impedance matching, reflection loss, absorption bandwidth, attenuation constant, and other parameters. These macroscopic performance indicators are the core parameters for evaluating the microwave absorption performance of MAMs.^[^ ^37^, ^38^, ^39^ ^]^ Among these, impedance matching is a key design objective for reducing surface reflection and enhancing absorption efficiency, and reflection loss reflects the overall absorption intensity of the materials to the incident microwaves. The absorption bandwidth determines the effective operating frequency band of the MAMs, and the attenuation constant characterizes the energy dissipation rate of microwaves within the MAMs.^[^ ^40^, ^41^, ^42^ ^]^

Good impedance matching characteristics enable the incident microwaves to enter the MAMs as much as possible, thus providing the opportunity to be converted into thermal energy or other forms of energy to dissipate, achieving effective absorption of microwaves.^[^ ^43^, ^44^, ^45^ ^]^ Among these, the reflection coefficient *Γ* (Equation (1)) of microwaves at the interface of the two media can be expressed as

(1) $$\Gamma =\left(Z_{\mathrm{in}}-Z_{0}\right)/\left(Z_{\mathrm{in}}+Z_{0}\right)$$

(2) $$Z_{0}=\sqrt{\mu_{0}/\varepsilon_{0}}$$

(3) $$Z_{\mathrm{in}}=\sqrt{\mu_{r}/\varepsilon_{r}}$$

In the formula, *Z_0_* (Equation (2)) and *Z_in_* (Equation (3)) are respectively the impedance in free space and the inherent impedance of the MAMs. *µ_0_* and *ε_0_* are respectively the permeability and permittivity in free space, and *µ_r_* (Equation (4)) and *ε_r_* (Equation (5)) are respectively the complex permeability and complex permittivity of the MAMs. The expressions are as follows

(4) $$\varepsilon_{r}=\sqrt{\varepsilon^{\prime }-j\varepsilon^{\prime \prime }}$$

(5) $$\mu_{r}=\sqrt{\mu^{\prime }-j\mu^{\prime \prime }}$$

In the formula, ε′ and µ′ are the real parts of ε_*r*_ and µ_*r*_, representing the energy storage capacity of the electric field and the magnetic field.^[^ ^46^ ^]^ *ε′′* and *µ′′* are the imaginary parts of *ε_r_* and *µ_r_*, representing the electric field and magnetic field loss capacities of the materials.^[^ ^47^ ^]^ When the relative input impedance |*Z_in_*/*Z_0_* | = 1, the inherent impedance of the MAMs is consistent with the impedance in free space. That is, the incident microwaves can fully enter the interior of the MAMs, reducing surface reflection and facilitating further attenuation of the microwaves.^[^ ^48^, ^49^, ^50^ ^]^ Therefore, to achieve strong microwave absorption, |*Z_in_*/*Z_0_* | should be as close as possible to 1.

The absolute value of the delta function (|∆|, Equation (6)) represents the degree to which the microwave input impedance of the MAMs deviates from the optimal matching state.^[^ ^51^, ^52^, ^53^ ^]^ It is also often used to describe the impedance matching characteristics between materials and free space.^[^ ^54^, ^55^, ^56^ ^]^ It is generally considered that the impedance matching is good when |*∆*| is less than 0.4. The *K* and *M* can be calculated through electromagnetic parameters.

(6) $$\left|\;\Delta |=\right|\sinh^{2}\left(\mathrm{Kfd}\right)-M|$$

*tanδ_ε_* (Equation (7)) and *tanδ_µ_* (Equation (8)) are respectively the tangent values of the dielectric loss angle and the magnetic loss angle, and are generally regarded as the evaluation of the dielectric loss and magnetic loss capabilities of MAMs to microwaves. The expressions are as follows:

(7) $$\tan\delta_{\varepsilon }=\varepsilon^{\prime \prime }/\varepsilon^{\prime }$$

(8) $$\tan\delta_{\mu }=\mu^{\prime \prime }/\mu^{\prime }$$

Therefore, the balance between dielectric loss and magnetic loss is the key to the strength of microwave attenuation capacity.^[^ ^57^, ^58^, ^59^ ^]^ The reflection loss (RL, Equations (9) and Equations (10)) and the effective absorption band (EAB) of MAMs are important criteria for evaluating the absorption performance of the materials. The RL can be expressed as:

(9) $$\mathrm{RL}=20\mathrm{lg}\left|\frac{Z_{\mathrm{in}}-Z_{0}}{Z_{\mathrm{in}}+Z_{0}}\right|$$

(10) $$Z_{\mathrm{in}}=Z_{0}{\left(\frac{\mu_{r}}{\varepsilon_{r}}\right)}^{\frac{1}{2}}\tanh\left[j\left(\frac{2\pi \mathrm{fd}}{c}\right){\left(\mu_{r}\varepsilon_{r}\right)}^{\frac{1}{2}}\right]$$

In the formula, *f* and *c* represent the frequency of microwaves and the speed of light respectively, and *d* is the thickness of the MAMs.^[^ ^60^ ^]^ In practical applications, when RL is −10 dB, the microwave absorption capacity of MAMs can reach 90%.^[^ ^61^ ^]^ Generally, the frequency band when RL is less than −10 dB is defined as EAB.^[^ ^62^ ^]^ Under less packing load and thinner absorption thickness, a smaller RL and a wider EAB indicate better absorption performance.

According to the theory of microwave transmission, the attenuation coefficient *α* (Equation (11)) is usually used to characterize the attenuation of microwaves in MAMs, and its expression is as follows:

(11) $$\begin{array}{ccc} \alpha & = & \sqrt{2}\pi f/c\\ & & \times \sqrt{\left(\mu^{\prime \prime }\varepsilon^{\prime \prime }-\mu^{\prime }\varepsilon^{\prime }\right)+\sqrt{{\left(\mu^{\prime \prime }\varepsilon^{\prime \prime }-\mu^{\prime }\varepsilon^{\prime }\right)}^{2}+{\left(\mu^{\prime }\varepsilon^{\prime \prime }+\mu^{\prime \prime }\varepsilon^{\prime }\right)}^{2}}} \end{array}$$

The larger the *α* value is, the stronger the microwave attenuation ability of the MAMs will be.^[^ ^47^ ^]^ Higher *ε′′* and *µ′′* as well as smaller *ε′* and *µ′* can effectively promote the attenuation capacity of the material. To achieve the ideal microwave absorption effect, the electromagnetic parameters of MAMs determine the impedance matching of the materials and also affect the attenuation characteristics of the materials. When designing MAMs, one cannot merely consider a single characteristic but needs to comprehensively consider impedance matching and attenuation capacity.

### 2.2 Influencing Factors for Low‐Frequency Microwave Absorption Performance

The key factors influencing the low‐frequency microwaves absorption performance mainly include the matching thickness of the materials, the microstructure size, and the anisotropic characteristics.

Typically, the frequency corresponding to the minimum reflection loss shifts toward the lower frequency as the material matching thickness (*d*) increases. This result can be explained by interference theory.^[^ ^63^, ^64^, ^65^ ^]^ When the relationship between the material matching thickness and the incident microwave wavelength (*λ*) satisfies Equation (12), the curve exhibits a peak.

(12) $$d=\left(2n+1\right)\lambda /4=\left(2n+1\right)\lambda_{0}/4\sqrt{\mu_{r}\varepsilon_{r}}\;\left(n=0,1,2,3\text{…}\right)$$

In the formula, *λ* and *λ_0_* are the wavelengths in MAMs and in free space, respectively, where *n* is an integer. If the thickness *d* of the MAMs is exactly an odd multiple of a quarter wavelength (*λ*/4), the phase difference between the emergent microwaves and the incident microwaves is exactly 180°. At this point, they completely cancel each other out due to the destructive interference principle, thereby attenuating the total reflected microwaves. This is known as the quarter‐wavelength theory.^[^ ^66^ ^]^ Therefore, the design of the matching thickness of MAMs is also one of the ways to adjust the low‐frequency microwave absorption performance.

The effective absorption frequency band has a mutual relationship with the microscopic size of the material, and its specific size selection is closely related to the skin depth, single domain size, and quantum size effect.^[^ ^67^, ^68^, ^69^ ^]^ For the frequency band of 8 GHz and below, the skin depth of the absorption unit is 4.5 µm. For magnetic materials with different crystal structures, the size of a single domain varies slightly, typically ranging from 15 to 25 nm.^[^ ^70^, ^71^, ^72^ ^]^ When the particle size continuously decreases to below the size of a single domain, the spin is increasingly affected by thermal fluctuations. And the material system becomes superparamagnetic, which is not conducive to microwave absorption.^[^ ^73^, ^74^, ^75^ ^]^ When the size of the absorption unit is the same as the energy of the microwave quantum, the absorption unit will cause additional resonant absorption to the incident microwaves. Among them, the single domain size that undergoes resonant absorption at 1—8 GHz is ≈10 to 25 nm.

Additionally, another way to enhance the low‐frequency microwave absorption of materials is to strengthen the magnetic crystal or shape anisotropy for the materials through microstructure regulation or component directional arrangement. Anisotropy significantly alters the electromagnetic parameter tensor of the material, thereby enhancing the resonance response capability in the low‐frequency band through the intrinsic frequency regulation mechanism.^[^ ^76^, ^77^, ^78^ ^]^ This characteristic enables the equivalent wavelength of the material to be effectively reduced at the same target absorption frequency, thereby breaking through the size limitation of traditional low‐dimensional absorption units (unit sizes usually need to be close to *λ*/4). For instance, by constructing layered anisotropic metamaterials, the unit size can be expanded to the sub‐wavelength scale (below *λ*/10), while maintaining efficient absorption and dissipation of low‐frequency microwaves. This ‘small size‐wide bandwidth‐low frequency absorption’ synergistic design significantly enhances the application flexibility of materials. On the other hand, the unique morphology of low‐dimensional magnets (fibrous or sheet‐like) induces significant local field enhancement effects.^[^ ^79^, ^80^, ^81^ ^]^ High‐intensity magnetic polarization regions can form at the fiber tip or at the sheet edge. This is due to sudden changes in geometric curvature and non‐uniform distributions of charge/spin density (tip effect), as well as uncompensated magnetic moments caused by hanging bonds of boundary atoms (edge effect).^[^ ^82^ ^]^ The spatial gradient of this local magnetic field will drive the long‐range magnetic coupling between adjacent structures. This includes dipole–dipole interaction or exchange coupling, thereby constructing a 3D magnetic coupling network throughout the materials, ultimately significantly improving magnetic loss capability.

In conclusion, when designing low‐frequency microwave materials, low‐dimensional strongly magnetic metallic materials with a single domain of over 15 nm and an absorption unit of less than 4.5 µm recommended to select. Then, magnetic metallic materials could be combined with dielectric loss‐type materials to prepare composites. Further, through component adjustment and structural design, impedance matching could be enhanced while achieving multiple loss coordination.

## 3 Material Systems for Low‐Frequency Microwave Absorption Composites

### 3.1 Carbon‐Based Composites

Carbon‐based materials (such as carbon fiber, graphene, and carbon nanotubes) have attracted significant attention due to the outstanding comprehensive performance in mechanics (high strength, high modulus, and light weight), thermology (high thermal conductivity and high thermal stability), and electricity (adjustable electrical conductivity).^[^ ^83^, ^84^, ^85^ ^]^ On the other hand, carbon‐based materials, when used alone, are more likely to achieve impedance matching in the high‐frequency band, while impedance mismatch often occurs in the low‐frequency band. This leads to the widespread problem of narrow absorption low‐frequency bands and weak low‐frequency microwave absorption performance. Therefore, they are often combined with other materials to introduce multiple loss mechanisms, improve impedance matching, and further enhance low‐frequency microwave absorption performance.^[^ ^86^, ^87^, ^88^ ^]^

#### 3.1.1 Carbon Fiber Composites

Carbon fiber (CF), as a prominent representative of 1D carbon‐based materials, has attracted extensive attention in the field of microwave absorption due to its low density, high aspect ratio, excellent strength, good dielectric properties, and corrosion resistance.^[^ ^89^, ^90^, ^91^ ^]^

**Figure 2:**
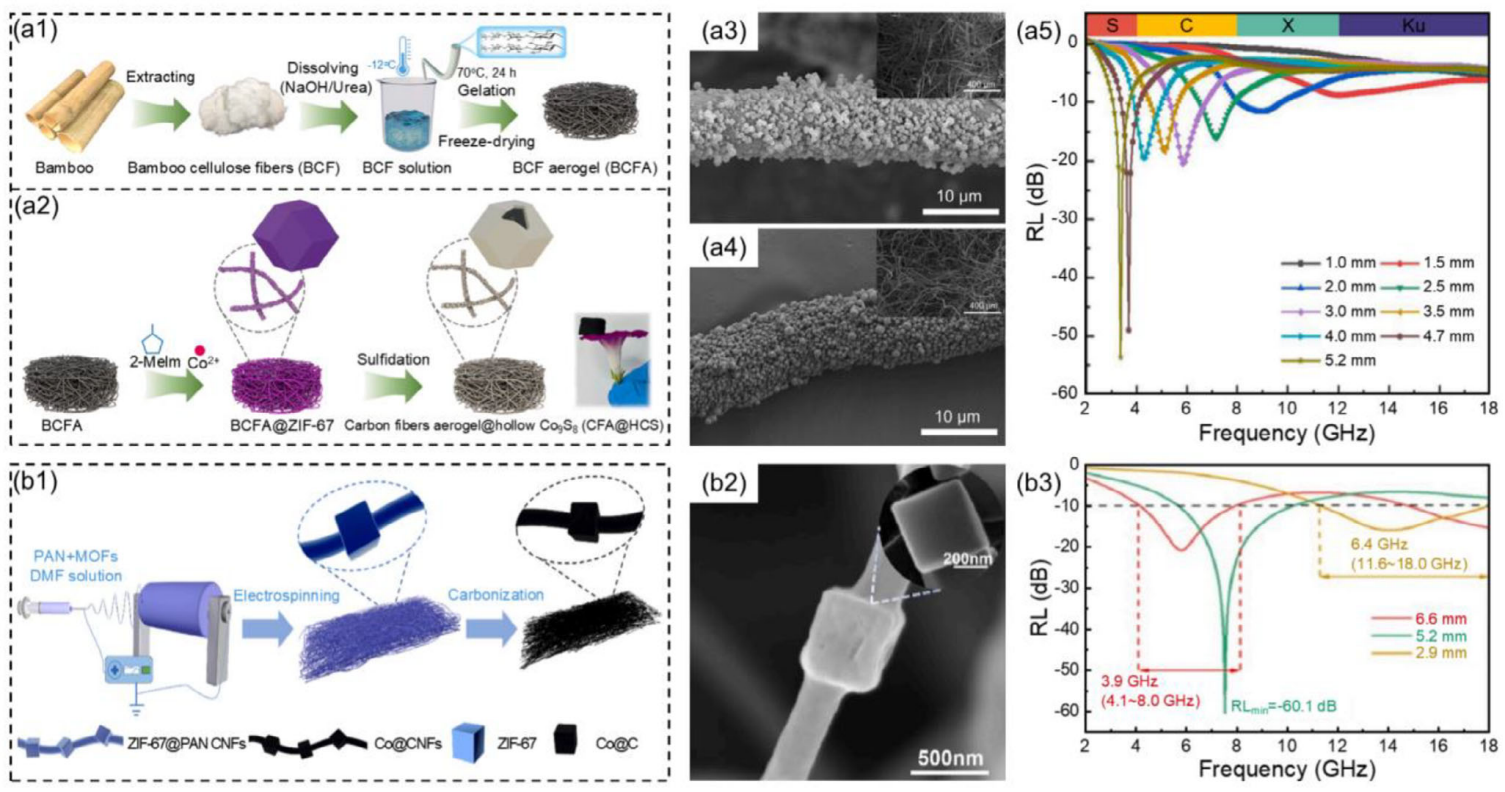
a1,a2) Schematic illustration of the preparation process for BCFA and CFA@HCS heterostructure MAMs, a3,a4) SEM images of BCFA@ZIF‐67 and CFA@HCS, a5) reflection loss values of CFA@HCS at corresponding thicknesses.^[^ ^92^ ^]^ Copyright 2023, Elsevier, b1) Schematic illustration of the fabrication process, b2) SEM images, and b3) reflection loss values at corresponding thicknesses of Co@CNFs.^[^ ^93^ ^]^ Copyright 2024, Elsevier.

Guo et al.^[^ ^92^ ^]^ first prepared bamboo cellulose fibers aerogel (BCFA) by cotton cellulose dissolution method (**Figure** **2**a1). Further, the heterostructure CFA@HCS with 3D interconnected BCFA as the framework supported with hollow cobalt sulfide was synthesized using the solvothermal method and continuous annealing process (Figure 2a2–a4). The microstructure and composition in CFA@HCS produced high dielectric polarization effect and conduction loss, resulting in a high low‐frequency microwave absorption effect. Additionally, hollow cobalt sulfide played a crucial role in regulating impedance matching and provided a large number of channels for multiple reflections and scattering of incident microwaves. When CFA@HCS was blended with paraffin, with the mass fraction was 15 wt.% and the thickness was 5.2 mm for CFA@HCS low‐frequency MAMs, the *RL*_min_ was −53.6 dB and the low‐frequency EAB was 0.7 GHz (3.0–3.7 GHz, Figure 2a5). Furthermore, when the angle of incidence *θ* = −60°, the minimum reduction of the radar cross section (RCS) was −22.6 dB m^2^. Wang et al.^[^ ^93^ ^]^ prepared a lightweight and flexible Co@C carbon nanofiber films (Co@CNFs) heterostructure MAMs by co‐electrospinning synthesis (Figure 2b1,b2). The results showed that when Co@CNFs was blended with paraffin, the mass fraction was 7.5 wt.% and the thickness was 5.2 mm for Co@CNFs low‐frequency MAMs, the *RL*_min_ was −60.1 dB, and the low‐frequency EAB was 2.3 GHz (5.7–8.0 GHz, Figure 2b3). Continuous carbon nanofibers provided broadband microwave attenuation capability, and the introduced MOF‐derived Co further enhanced the low‐frequency microwave absorption performance by inducing magnetic loss, constructing rich interfaces, and regulating electrical conductivity.

#### 3.1.2 Graphene Composites

**Figure 3:**
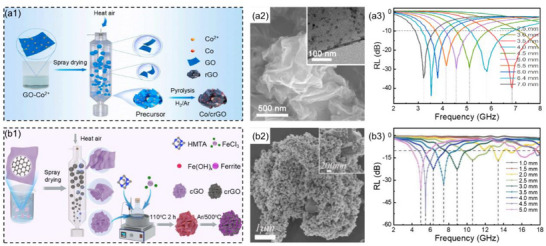
a1) Schematic illustration of the preparation process, a2) SEM with TEM images, and a3) the reflection loss values at the corresponding thicknesses for Co/crGO.^[^ ^94^ ^]^ Copyright 2023, Elsevier, b1) Schematic illustration of the fabrication process, b2) SEM image, and b3) reflection loss values at corresponding thicknesses for Fe_3_O_4_@crGO.^[^ ^95^ ^]^ Copyright 2025, ACS.

Reduced graphene oxide (rGO) is a unique 2D material with characteristics such as a large specific surface area, numerous defects, and localized Fermi levels, which easily induce interfacial polarization and surface scattering, thereby enhancing the microwave absorption performance in the low‐frequency band. Xu et al.^[^ ^94^ ^]^ prepared Co nanocrystals anchored on wrinkled spherical reduced graphene oxide (Co/crGO, **Figure** **3**a1,a2) through a stepwise spray‐drying‐sintering process. When the amount of GO was 500 mg, the dosage of Co(NO_3_)_2_·6H_2_O was 1 mmol, and the thickness of the Co/crGO low‐frequency MAMs was 6.4 mm, the *RL*_min_ was −43.3 dB and the low‐frequency EAB was 0.9 GHz (3.2–4.1 GHz, Figure 3a3). This was mainly attributed to the fact that the wrinkled spherical structure generates large voids and rich heterogeneous interfaces, which jointly promoted dielectric polarization loss and impedance matching. Furthermore, the addition of magnetic components promoted increases in magnetic loss capacity while preventing the agglomeration of graphene flakes and magnetic nanoparticles, ensuring the appropriate exposure of polar functional groups on graphene.

Liu et al.^[^ ^95^ ^]^ prepared wrinkled monolayer graphene oxide spheres by the spray‐drying method. Subsequently, iron hydroxide nanocrystals were synthesized in graphene oxide spheres by a simple oil‐bath heating method. Further annealing in an argon atmosphere prepared heterostructure MAMs with Fe_3_O_4_ magnetic crystals loaded onto reduced graphene oxide spheres (Fe_3_O_4_@crGO, Figure 3b1,b2). This wrinkled structure provided a large number of interfaces for the high‐loading Fe_3_O_4_ nanocrystals, effectively avoiding severe agglomeration. This not only reduced the permittivity but also enhanced the permeability of the material, thereby optimizing the impedance matching of MAMs in the low‐frequency band. When the amount of GO was 90 mg, the amount of FeCl_3_·6H_2_O was 3 mmol, and the thickness of Fe_3_O_4_@crGO low‐frequency MAMs was 4.5 mm, the *RL*_min_ was −52.3 dB, and the low‐frequency EAB was 2.3 GHz (4.6–6.9 GHz, Figure 3b3). Because of the excellent dielectric loss for reduced graphene oxide, the magnetic loss of Fe_3_O_4_ magnetic crystals, the polarization loss of heterogeneous interfaces, and the multiple reflections and scattering of microwaves within the 3D structure jointly enhanced the low‐frequency microwave attenuation capacity of MAMs.

#### 3.1.3 Carbon Nanotube Composites

Carbon nanotubes (CNT) have become one of the MAMs with development potential due to their advantages such as controllable morphological structure, stable chemical properties, and adjustable electrical conductivity.^[^ ^96^, ^97^, ^98^ ^]^

**Figure 4:**
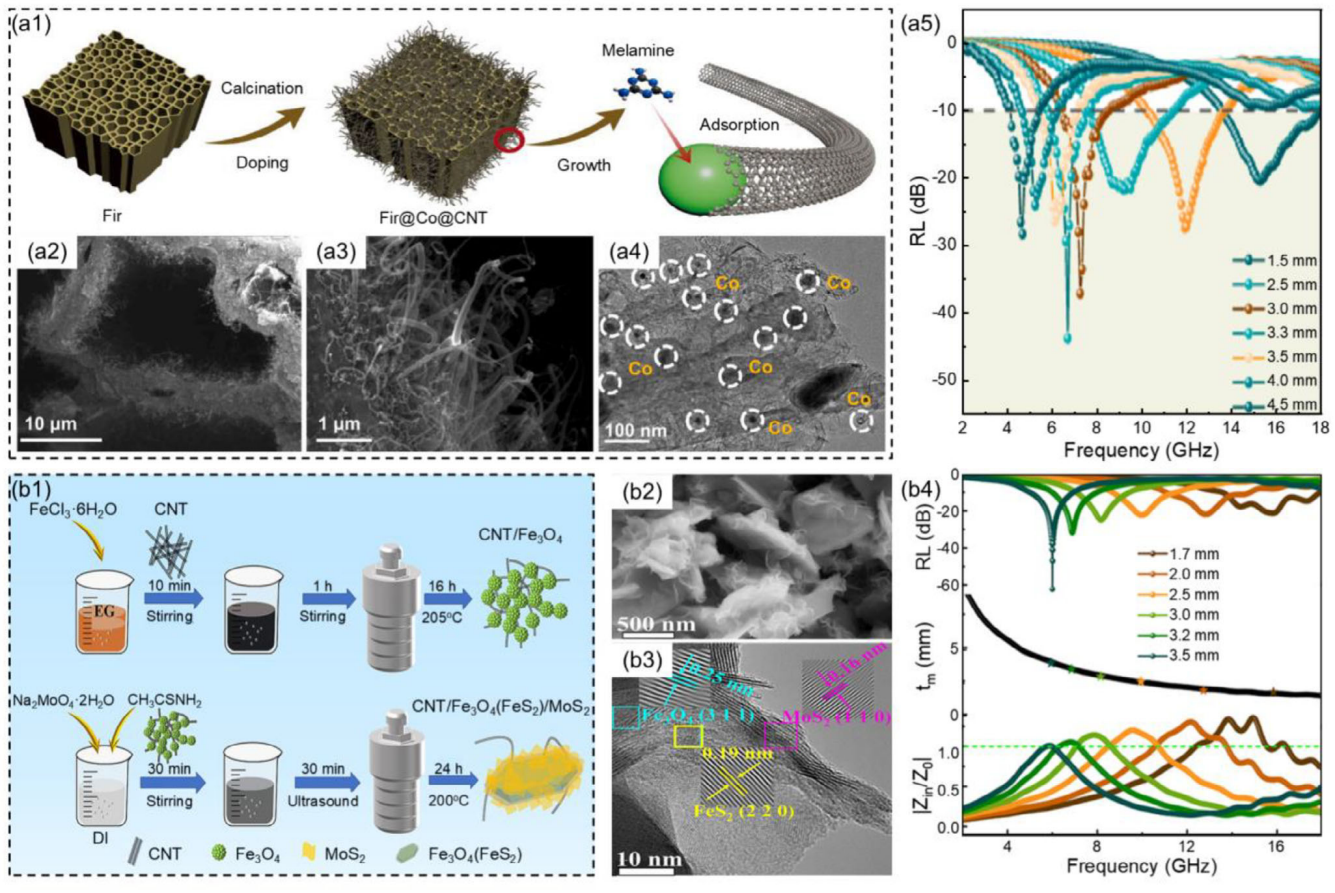
a1) Schematic illustration of the preparation process, a2,a3) SEM images, a4) TEM image, a5) corresponding reflection loss values at different thicknesses of Fir@Co@CNT.^[^ ^99^ ^]^ Copyright 2024, Elsevier, b1) Schematic illustration of the fabrication process, b2) TEM image, b3) HRTEM image, b4) corresponding reflection loss values, matching thicknesses, as well as |*Z_in_/Z_0_* | values at different thicknesses for CNT/Fe_3_O_4_(FeS_2_)/MoS_2_.^[^ ^100^ ^]^ Copyright 2024, ACS.

Yang et al.^[^ ^99^ ^]^ used wood as raw material and grew nitrogen‐doped CNTs in situ on porous wood‐derived carbon via chemical vapor deposition, then loaded magnetic metal Co nanoparticles to prepare Fir@Co@CNT composites (**Figure** **4**a1–a4). By adjusting the amount of CNT, precise control over the effects of polarization loss and conduction loss in Fir@Co@CNT composites was achieved. When the mass ratio of melamine to Fir@Co was 5:1 and the thickness of the Fir@Co@CNT composites was 3.3 mm, the *RL*_min_ of the composites was −43.0 dB and the low‐frequency EAB was 1.9 GHz (5.8–7.7 GHz, Figure 4a5). This was primarily due to the 3D conductive network formed by biomass porous carbon and the CNT generated on its surface, which significantly enhanced the transmission and loss capacity of microwave absorption and improved impedance matching. Additionally, the regular hollow channel facilitated the entry of microwaves into the MAMs and induced multiple scattering, thereby enhancing the absorption of incident microwaves. Xiang et al.^[^ ^100^ ^]^ synthesized CNT/Fe_3_O_4_(FeS_2_)/MoS_2_ composites by solvothermal and hydrothermal methods (Figure 4b1–b3). By controlling the amount of sulfur source (thioacetamide), the tunability of microwave absorption frequency from the S band to the C band was achieved. When the amount of thioacetamide was 0.32 g, the CNT/Fe_3_O_4_(FeS_2_)/MoS_2_ composites exhibited excellent *RL*_min_ and EAB, and the thickness was 3.5 mm, the *RL*_min_ was −62.3 dB and the low‐frequency EAB was 2 GHz (5–7 GHz, Figure 4b4). This was mainly attributed to the multiple heterogeneous interfaces in the CNT/Fe_3_O_4_(FeS_2_)/MoS_2_ composites, including CNT/Fe_3_O_4_, Fe_3_O_4_/FeS_2_, FeS_2_/MoS_2_, and MoS_2_/air, which resulted in uneven charge distribution in multiple regions and enhanced interface polarization. Additionally, the asymmetric charge distribution on functional groups or heteroatoms on CNT, Fe_3_O_4_, FeS_2_, and MoS_2_ also caused dipole polarization. Meanwhile, the large specific surface area of MoS_2_ formed a more abundant conductive network, extending the electron migration path and improving conductive loss.

### 3.2 Magnetics‐Based Composites

Magnetic loss type MAMs possess high saturation magnetization strength and magnetic anisotropy, enabling their natural resonance frequency to surpass the Snoek limit.^[^ ^101^, ^102^ ^]^ This confers a unique advantage in achieving strong microwave absorption in the S and C bands. Traditional magnetic loss type MAMs include iron, cobalt, and nickel (Fe, Co, and Ni) metals and carbonyl iron (CIP) with different morphologies, which exhibit microwave absorption performance. However, they suffer from drawbacks such as a tendency to agglomerate, poor impedance matching, and suboptimal microwave attenuation efficiency.^[^ ^103^, ^104^, ^105^ ^]^ Compared to traditional magnetic loss type materials, CIP composite systems and binary magnetic alloys (such as FeCo, FeNi, and CoNi) exhibit excellent impedance matching characteristics in the low‐frequency band. By adjusting the chemical composition, morphology, and size of binary magnetic alloys through structural design, the natural resonance in the S and C bands can be significantly enhanced, potentially achieving efficient improvements in absorption performance in the low‐frequency range.^[^ ^106^, ^107^, ^108^ ^]^

#### 3.2.1 Carbonyl Iron Composites

**Figure 5:**
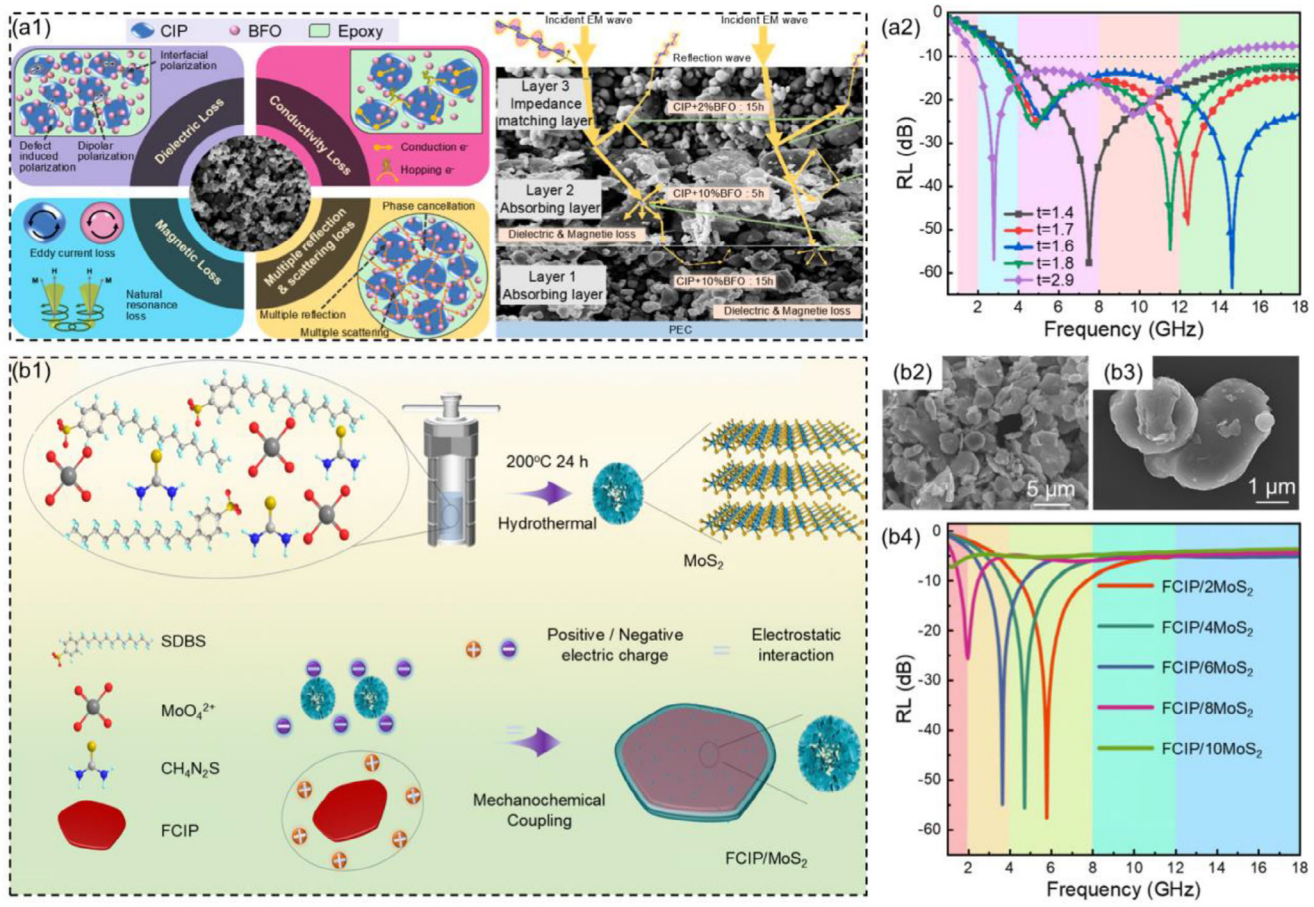
a1) Microwave absorption mechanisms as well as SEM images and a2) corresponding reflection loss values at different thicknesses of three‐layer CIP/BFO composite materials.^[^ ^111^ ^]^ Copyright 2023, Elsevier, b1) Schematic illustration of the preparation process and b2,b3) SEM images for FCIP/MoS_2_ composites, b4) reflection loss values composite materials at different MoS_2_ contents for FCIP/MoS_2_.^[^ ^112^ ^]^ Copyright 2024, RSC.

CIP composites can utilize surface spin pinning and nanocrystalline grain boundary scattering to enhance natural resonance and localized eddy current losses. Additionally, the shape anisotropy of plate‐like CIP materials can overcome the Snoek limit, enabling low‐frequency microwave absorption through multi‐scale magnetic loss coordination.^[^ ^109^, ^110^ ^]^ Singh et al.^[^ ^111^ ^]^ prepared carbonyl iron/barium ferrite (CIP/BFO) with different ball milling times and different BFO mass fractions by the high‐energy ball milling method, and optimized the design of three‐layer CIP/BFO composites. The multi‐layer CIP/BFO composites effectively increased the absorption bandwidth and reduced the thickness of the absorbing coating through the synergistic effect of multiple microwave absorption mechanisms such as eddy current loss, conductive loss, and multiple interface polarization (**Figure** **5**a1). When the mass fraction of the upper layer BFO was 2 wt.% and ball milling was 15 h, the mass fraction of the middle layer BFO was 10 wt.% and ball milling was 5 h, the mass fraction of the lower layer BFO was 10 wt.% and ball milling was 15 h, the three‐layer CIP/BFO composites with the thickness of 2.9 mm achieved the *RL*_min_ of −57.0 dB and the EAB of 11.9 GHz (1.7–13.6 GHz, Figure 5a2). Wu et al.^[^ ^112^ ^]^ synthesized flake carbonyl iron powder/molybdenum disulfide (FCIP/MoS_2_) composites by the hydrothermal method combined with ball milling technology (Figure 5b1–b3). The introduction of MoS_2_ not only significantly optimized the impedance matching but also enabled flexible regulation of the microwave absorption frequency band of the composites, extending the coverage range from the X band to the lower frequency L band. When the mass fraction of MoS_2_ was 2 wt.% and the thickness of the FCIP/2MoS_2_ composites was 2.4 mm, the *RL*_min_ was −57.6 dB and EAB was 3.3 GHz (4.5–7.8 GHz, Figure 5b4). When the mass fraction of MoS_2_ increased to 8 wt.% and the thickness of the FCIP/8MoS_2_ composites was 4.5 mm, the *RL*_min_ was −25.7 dB and the low‐frequency EAB was 0.9 GHz (1.6–2.5 GHz, Figure 5b4).

#### 3.2.2 Binary Magnetic Alloy Composites

Binary magnetic alloy composites lay the foundation for low‐frequency magnetic loss with high saturation magnetization strength, and drive low‐frequency natural resonance through controllable anisotropy (magnetic crystal and shape), to become a new generation of low‐frequency MAMs that combine customizable frequency bands, ultra‐thin broadband absorption, and environmental robustness.^[^ ^113^ ^]^

**Figure 6:**
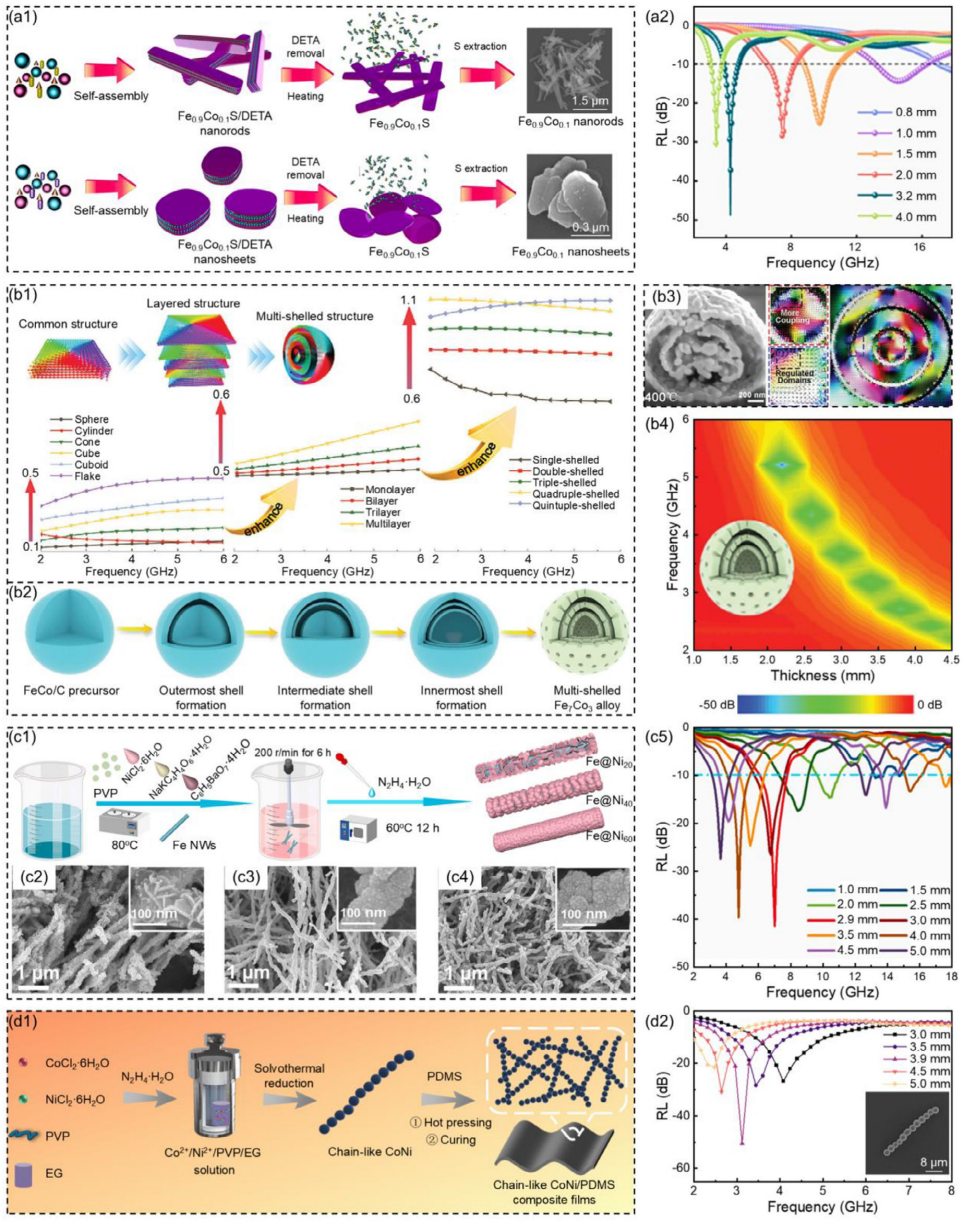
a1) Schematic illustration of the preparation process and SEM images for rod‐like and sheet‐like Fe_0.9_Co_0.1_ nanocomposites, a2) reflection loss values for sheet‐like Fe_0.9_Co_0.1_ nanocomposites at corresponding thicknesses.^[^ ^114^ ^]^ Copyright 2024, Elsevier, b1) Micromagnetism simulation of FeCo alloys with different morphologies, b2) reduction process of ferromagnetic multi‐shell FeCo alloys, b3) SEM images, intershell magnetic interaction, microscopic characterization, and micromagnetism simulation of stray field coupling for the three‐shell structure of FeCo alloys, b4) 2D color‐mapping of reflection loss for FeCo alloys.^[^ ^115^ ^]^ Copyright 2024, Wiley‐VCH, c1) Schematic illustration of the fabrication process, c2–c4) SEM images, and c5) reflection loss values at corresponding thicknesses for Fe@Ni_X_ nanowires.^[^ ^116^ ^]^ Copyright 2024, Elsevier, d1) Schematic illustration of the preparation process and d2) the reflection loss values at the corresponding thickness for chain‐like CoNi.^[^ ^117^ ^]^ Copyright 2024, Wiley‐VCH.

Wang et al.^[^ ^114^ ^]^ designed and fabricated 1D (rod‐like) and 2D (sheet‐like) Fe_0.9_Co_0.1_ nanocomposites by using the organic template method combined with the constrained transformation strategy (**Figure** **6**a1). The influence of anisotropy on the low permeability of monodisperse Fe_0.9_Co_0.1_ nanocomposites was systematically investigated. The results showed that when the thickness of the sheet‐like Fe_0.9_Co_0.1_ nanocomposites was 3.2 mm, the *RL*_min_ was −48.7 dB and the low‐frequency EAB was 0.9 GHz (3.9–4.8 GHz, Figure 6a2). The high saturation magnetization and Snoek limit of sheet‐like Fe_0.9_Co_0.1_ contributed to enhancing the microwave absorption performance. The large specific surface area facilitated the accumulation of charges at the heterogeneous interfaces and interface polarization, promoting multiple reflections and scattering of microwaves within the sheet‐like Fe_0.9_Co_0.1_. Che et al.^[^ ^115^ ^]^ simulated the low‐frequency magnetic spectra of FeCo alloys with different shapes, structures, and interlayer spacings, and screened out FeCo alloys with the most excellent microwave absorption performance in the frequency range of 2 to 6 GHz (Figure 6b1). Subsequently, based on the simulation results, the multi‐shell FeCo alloy with a controllable number of shells was developed using the isothermal diffusion method, and the formation mechanism of the shells was analyzed by in situ transmission electron microscopy (Figure 6b2). The anomalous interlayer magnetic domain regulation behavior achieved through interlayer magnetic interactions, effectively overcoming the limitations of spherical structures in the low‐frequency Snoek limit, significantly enhancing the low‐frequency magnetic permeability and microwave absorption capacity (Figure 6b3). When the number of shell layers of the FeCo alloy was three and the material thickness was 2.2 mm, the *RL*_min_ was −42.1 dB and the low‐frequency EAB was 1.6 GHz (4.4–6 GHz, Figure 6b4). Because the interlayer spacing of the three‐shell structure FeCo alloy was reduced, enhancing the effective magnetic interaction between the shells. The unique middle shell layer could act as a bridge between the outermost shell and the innermost shell, significantly enhancing the low‐frequency magnetic response of the three‐shell structure FeCo alloy, thereby improving its microwave absorption capacity.

Li et al.^[^ ^116^ ^]^ prepared 1D core–shell Fe@Ni_X_ nanowires by liquid‐phase reduction (Figure 6c1–c4). When the mass ratio of Fe to Ni was 6:4 and the thickness of Fe@Ni_40_ nanowires low‐frequency MAMs was 2.9 mm, the *RL*_min_ was −41.6 dB and the low‐frequency EAB was 1.7 GHz (6.1–7.8 GHz, Figure 6c5). When microwaves were incident vertically, the minimum attenuation of RCS is −9.4 dB m^2^. This was mainly attributed to the strong magnetic coupling brought about by the bimetallic composition, which enhanced the resonance energy level. Additionally, the excellent 1D core–shell structure improved the polarization loss efficiency and broadened the low‐frequency absorption bandwidth. In our previous work, Gu et al.^[^ ^117^ ^]^ used polyvinylpyrrolidone as the shape‐directing agent and prepared anisotropic chain‐like CoNi low‐frequency MAMs through the solvothermal reduction method (Figure 6d1). When the thickness of the chain‐like CoNi low‐frequency MAMs was 3.9 mm, the *RL*_min_ was −50.5 dB and the low‐frequency EAB was 1.1 GHz (2.6–3.7 GHz, Figure 6d2). This was mainly attributed to the excellent shape anisotropy and strong magnetic coupling effect of the chain‐like CoNi, which was conducive to enhancing the natural resonance of low‐frequency, thereby improving the low‐frequency microwave absorption performance. At the same time, these performances contributed to enhancing the magnetic loss capability and low‐frequency impedance matching for chain‐like CoNi.

### 3.3 Polymer‐Based Composites

Polymer‐based microwave absorption composites, due to the advantages such as strong design flexibility, tunable electromagnetic parameters, and convenient molding processing, can meet the development trends of innovative MAMs toward lightweight, high efficiency, and novelty.^[^ ^118^ ^]^ Polymer‐based composites primarily consist of polymers and absorbers. Among them, the polymers serve as the carrier of the MAMs and act as the channel for transmitting microwaves, while the absorbers are responsible for absorbing microwaves.^[^ ^14^, ^119^, ^120^ ^]^ Based on the differences in structural design, polymer‐based microwave absorption composites can be classified into layered structure, hollow structure, and homogeneous structure, etc.^[^ ^121^, ^122^ ^]^ To meet the demands of different application fields for microwave absorption composites, the multi‐functionality of polymer‐based microwave absorption composites has become the key trend of innovative MAMs.

#### 3.3.1 Layered Composites

Layered polymer‐based microwave absorption composites can induce multiple reflections of microwaves within the composites, significantly enhancing the microwave absorption performance.^[^ ^123^, ^124^, ^125^ ^]^

**Figure 7:**
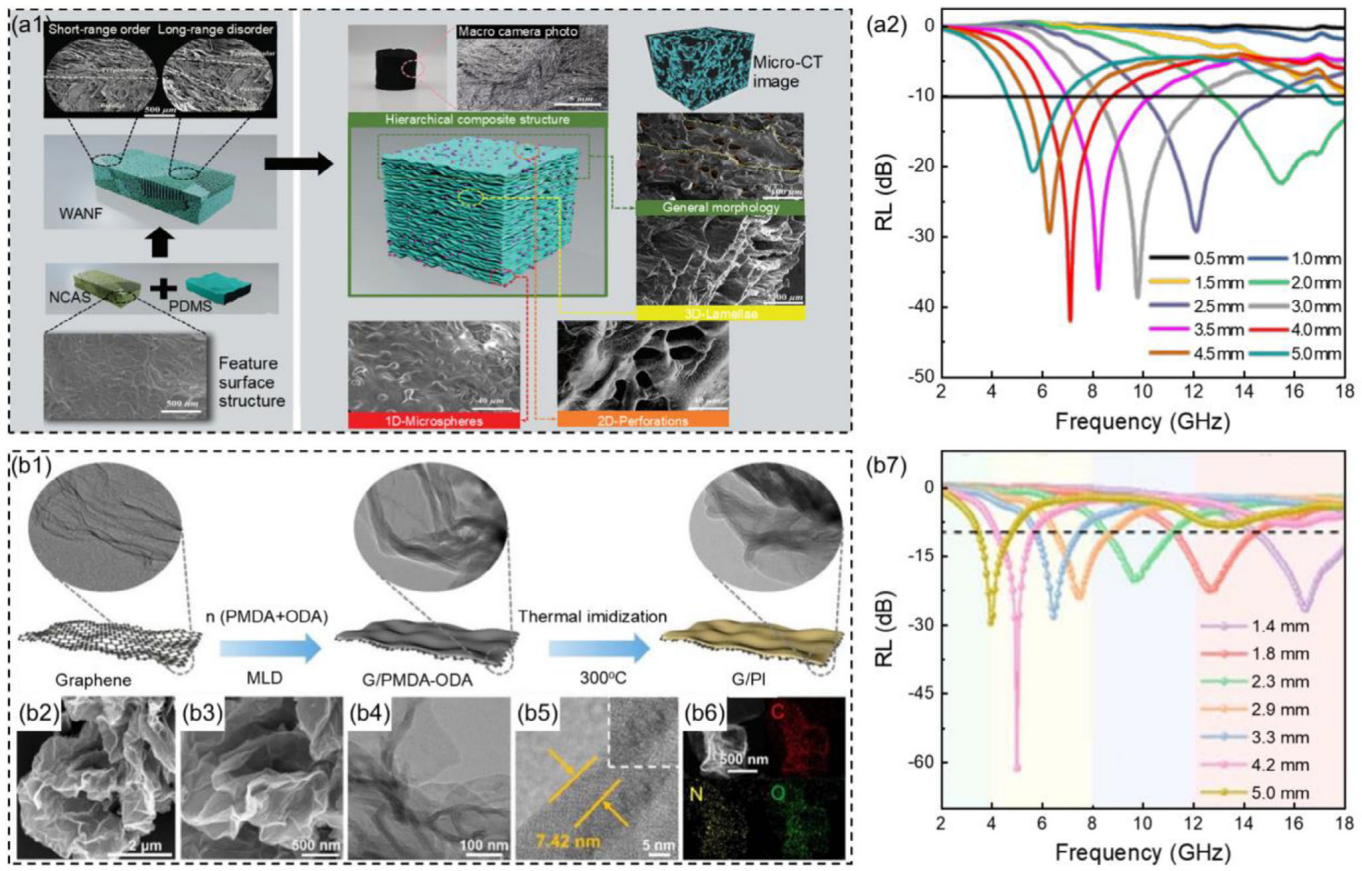
a1) SEM images and schematic illustration of the preparation process and a2) the reflection loss values at the corresponding thickness for WANF composites.^[^ ^126^ ^]^ Copyright 2023, Wiley‐VCH, b1) Schematic illustration of the fabrication process, b2,b3) SEM images, b4) TEM image, b5) HRTEM image, b6) EDS element distribution images, and b7) reflection loss values at corresponding thicknesses for G/PI composites.^[^ ^127^ ^]^ Copyright 2023, RSC.

Qiu et al.^[^ ^126^ ^]^ were inspired by the rough endoplasmic reticulum, and introduced GO and polydimethylsiloxane (PDMS) into CNT aerogels to construct the nano‐carbon foam (WANF, **Figure** **7**a1) with the unique layered composite structures (3D layered, 2D porous, and 1D microspheres). When the concentration of CNT was 16.67 mg mL^−1^, the mass ratio of GO to CNT was 2:3, and the thickness of the WANF composites was 4.0 mm, the *RL*_min_ was −40.1 dB and the low‐frequency EAB was 2.0 GHz (6.0–8.0 GHz, Figure 7a2). Furthermore, due to unique mechanisms such as vortex shedding, secondary reflection, and resonance, WANF exhibited excellent sound absorption performance, with a bandwidth exceeding 0.9 for the sound absorption coefficient reaching 4.75 kHz. Wang et al.^[^ ^127^ ^]^ uniformly deposited aromatic polyimide (PI) on the surface of graphene by molecular layer deposition (MLD) method and adjusted the number of deposition cycles, successfully preparing graphene/PI (G/PI) composites with excellent microwave absorption and thermal management performances (Figure 7b1–b6). Benefiting from excellent dielectric loss, appropriate impedance matching, and strong attenuation capacity, G/PI composites exhibited strong and wideband low‐frequency microwave absorption performance. When the MLD cycle was 40 times and the thickness of the G/PI composites was 4.2 mm, the *RL*_min_ was −60.7 dB and the low‐frequency EAB was 1.6 GHz (4.2–5.8 GHz, Figure 7b7). The RCS simulation results indicated that the G/PI composites significantly suppress strong scattering of microwaves.

#### 3.3.2 Hollow Composites

**Figure 8:**
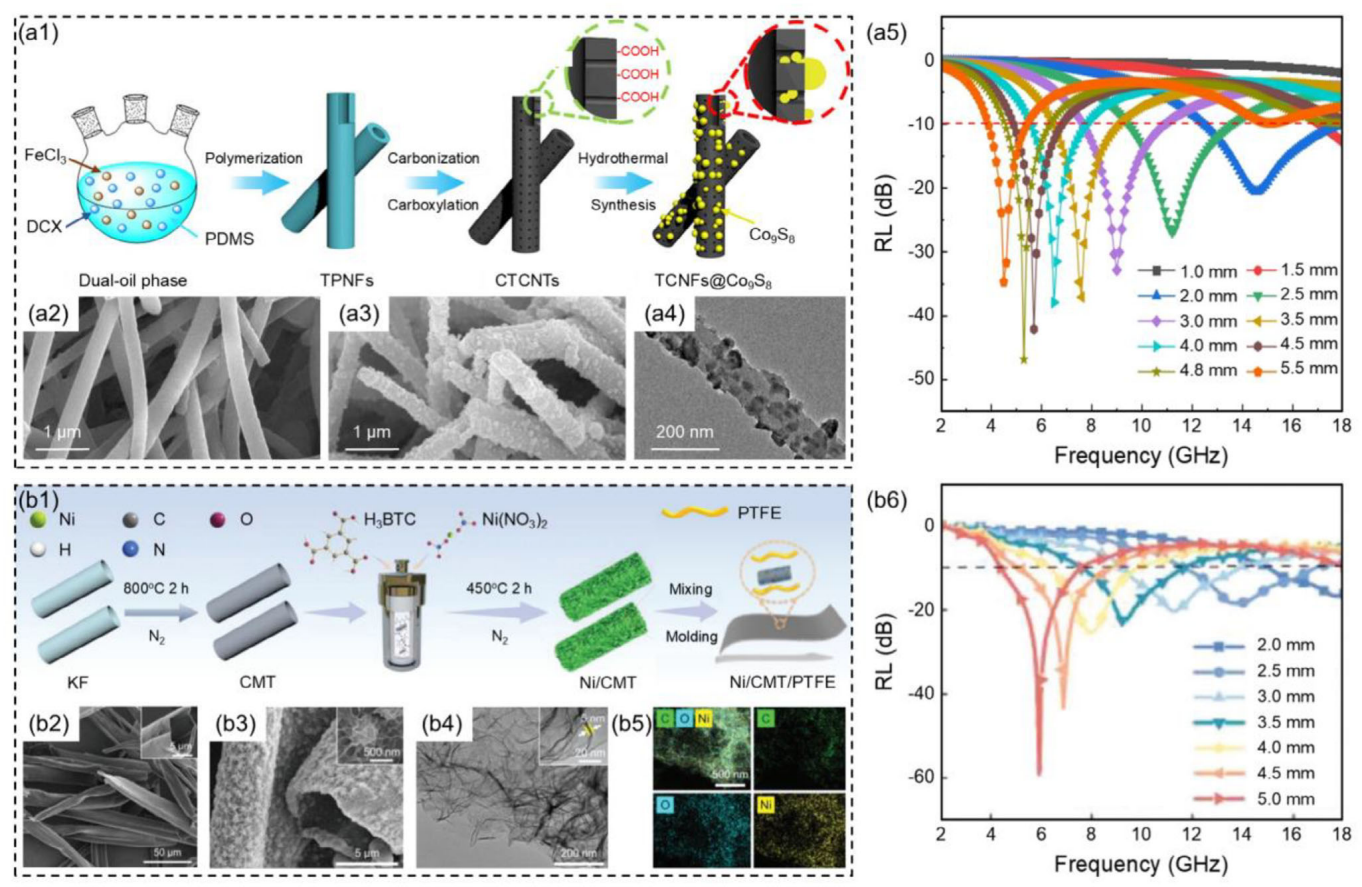
a1) Schematic illustration of the preparation process for CTCNFs/Co_9_S_8_ hybrid nanofibers, a2) SEM image of CTCNFs, a3,a4) SEM images and TEM image of CTCNFs/Co_9_S_8_ hybrid nanofibers, a5) reflection loss values of CTCNFs/Co_9_S_8_/PVDF composites at corresponding thicknesses.^[^ ^131^ ^]^ Copyright 2020, IOP Publishing, b1) Schematic illustration of the fabrication process for Ni/CMT/PTFE composite films, b2) SEM image of CMT, b3–b5) SEM image, TEM image and EDS element distribution images of Ni/CMT, b6) reflection loss values of Ni/CMT/PTFE composite films at the corresponding thickness.^[^ ^132^ ^]^ Copyright 2023, Wiley‐VCH.

Hollow polymer‐based microwave absorption composites can reflect microwaves multiple times due to the rich heterogeneous interfaces, thereby prolonging or delaying the microwave transmission path.^[^ ^128^, ^129^, ^130^ ^]^ Zhang et al.^[^ ^131^ ^]^ synthesized tubular polymer nanofibers (TPNFs) by restricting self‐condensation polymerization, and further transformed TPNFs into surface carboxylated tubular carbon nanofibers (CTCNFs) through carbonization and acidification treatments. Then CTCNFs/Co_9_S_8_ hybrid nanofibers were prepared by controllable growth of Co_9_S_8_ nanoparticles on CTCNFs by the hydrothermal method, finally composited with polyvinylidene fluoride (PVDF) to fabricate CTCNFs/Co_9_S_8_/PVDF composites (**Figure** **8**a1–a4). CTCNFs/Co_9_S_8_ hybrid nanofibers possessed abundant effective interfaces and defective dipoles, which effectively enhance the polarization effect. Through this strategy of enhancing dielectric loss, the microwave dissipation capacity of CTCNFs/Co_9_S_8_ hybrid nanofibers was significantly improved, demonstrating excellent low‐frequency microwave absorption performance. When the mass fraction of CTCNFs/Co_9_S_8_ hybrid nanofibers was 15 wt.% and the thickness of the CTCNFs/Co_9_S_8_/PVDF composites was 4.8 mm, the *RL*_min_ of the composite was −46.8 dB and the low‐frequency EAB was 1.9 GHz (4.5–6.4 GHz, Figure 8a5).

Wang et al.^[^ ^132^ ^]^ first prepared nickel/carbon microtubes (Ni/CMT) with a layered hollow structure, then combined them with a processable polytetrafluoroethylene (PTFE) matrix to construct the flexible Ni/CMT/PTFE composite films (Figure 8b1–b5). The large inner cavity of CMT in the Ni/CMT/PTFE composite films effectively improved impedance matching and provided an additional channel for microwave dissipation, while the layered magnetic nickel nanoparticles enhanced the synergy between the closed heterogeneous interfaces, achieving low‐frequency broadband and strong microwave absorption performance. When the mass fraction of Ni/CMT was 3 wt.% and the thickness of the Ni/CMT/PTFE composite films was 5 mm, the *RL*_min_ of the composite films was −59.1 dB and the low‐frequency EAB was 3.1 GHz (4.3–7.4 GHz, Figure 8b6). The CST simulation results further confirmed that the Ni/CMT/PTFE composite films possessed significant radar cross‐section reduction capabilities with −34.1 dB m^2^ at 60°. Additionally, the Ni/CMT/PTFE composite films exhibited excellent joule heating performance, hydrophobicity, and flame retardancy. Based on this, a novel patterned strain‐sensing sensor with high sensitivity was constructed, which could be applied in scenarios such as electromagnetic interference shielding, waterproofing as well as deicing, fire safety, and health monitoring.

#### 3.3.3 Homogeneous Composites

**Figure 9:**
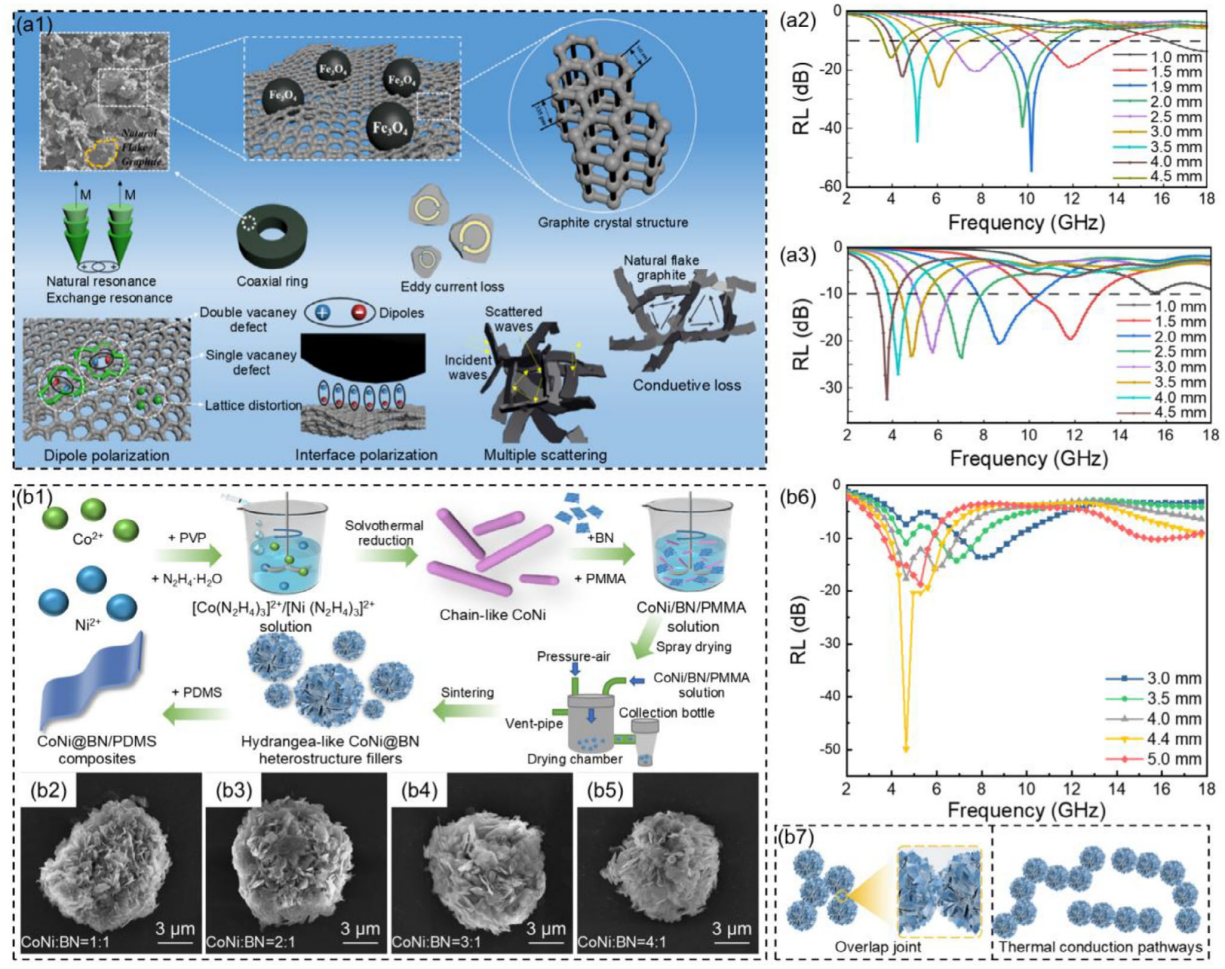
a1) SEM images and microwave absorption mechanisms of graphite/Fe_3_O_4_/phenolic resin/epoxy resin composites, a2,a3) reflection loss values of graphite/Fe_3_O_4_/phenolic resin/epoxy resin composites at corresponding thicknesses when the mass ratio of graphite to Fe_3_O_4_ is 4:3 and 5:2.^[^ ^134^ ^]^ Copyright 2023, Wiley‐VCH, b1) Schematic illustration of the preparation process of CoNi@BN/PDMS composites, b2–b5) SEM images of CoNi@BN for CoNi and BN at different mass ratios, b6) reflection loss values at corresponding thicknesses, and b7) thermal conductivity mechanisms of CoNi@BN/PDMS composites.^[^ ^135^ ^]^ Copyright 2024, Wiley‐VCH.

Homogeneous polymer‐based microwave absorption composites uniformly disperse MAMs in the polymer matrix. This method is simple to operate, low in cost, and can achieve multi‐functionality and large‐scale preparation, with broad industrial application prospects.^[^ ^133^ ^]^ Wu et al.^[^ ^134^ ^]^ prepared graphite/Fe_3_O_4_/phenolic resin/epoxy resin composites by selective laser sintering technology and epoxy resin vacuum impregnation process (**Figure** **9**a1). With the increase of the dosage of Fe_3_O_4_, the absorption peak of the composites showed a tendency to gradually shift from high frequency (12–18 GHz) to low frequency (2–8 GHz). When the mass ratio of graphite to Fe_3_O_4_ was 4:3 and the thickness of the graphite/Fe_3_O_4_/phenolic resin/epoxy resin composites was 1.9 mm, the *RL*_min_ of the composites was −54.8 dB and the high‐frequency EAB was 2.8 GHz (8.7–11.5 GHz, Figure 9a2). When the mass ratio of graphite to Fe_3_O_4_ was 5:2 and the thickness of the graphite/Fe_3_O_4_/phenolic resin/epoxy resin composites was 4.5 mm, the *RL*_min_ was −33.0 dB and the low‐frequency EAB was 0.8 GHz (3.4–4.2 GHz, Figure 9a3). By adjusting the amount of Fe_3_O_4_ used, it was possible to change the type of non‐homogeneous interfaces and impedance matching capabilities of the materials, enabling them to adapt more effectively to complex and variable microwave environments.

In our previous work, Gu et al.^[^ ^135^ ^]^ assembled chain‐like CoNi and sheet‐like BN into hydrangea‐like CoNi@BN heterostructure fillers using the spray‐drying‐sintering process. Then fillers were combined with PDMS to prepare low‐frequency microwave absorption and thermal conduction integrated CoNi@BN/PDMS composites (Figure 9b1–b5). The insulating BN coated the CoNi with high electrical conductivity and high saturation magnetization intensity, effectively reducing the mutual contact between CoNi. The permittivity of CoNi@BN/PDMS composites was reduced while maintaining excellent magnetic loss, resulting in superior impedance matching performance at low‐frequency. When the volume fraction of CoNi@BN was 44 vol% and the mass ratio of CoNi to BN was 3:1, the CoNi@BN/PDMS composites exhibited optimal low‐frequency microwave absorption performance and thermal conductivity. At the thickness of 4.4 mm, the *RL*_min_ was −49.9 dB and the low‐frequency EAB was 2.4 GHz (3.9–6.3 GHz), fully covering the n79 band (4.4–5.0 GHz) involved in 5G communications (Figure 9b, 6). The in‐plane thermal conductivity (*λ_∥_*) was 7.31 W m^−1^ K^−1^, ≈11.4 times higher than that of pure PDMS (0.64 W m^−1^ K^−1^). The improvement of thermal conductivity was attributed to the formation of effective thermal conduction pathways with BN‐BN overlaps (Figure 9b7), fully leveraging the thermal conductivity advantages of BN, thereby achieving integrated low‐frequency microwave absorption and thermal conductivity.

### 3.4 Ceramic‐Based Composites

To enhance the microwave absorption performance of ceramics, researchers mainly study polymer‐derived ceramics including silicon carbide (SiC), silicon carbide nitrogen (SiCN), silicon carbide oxygen (SiCO), and silicon boron carbon nitrogen (SiBCN) through methods such as composition and morphology design.^[^ ^136^, ^137^ ^]^ Additionally, the structural design based on metamaterials can overcome the limitations of material intrinsic properties, enabling efficient, broadband, or tunable absorptive performance in specific frequency bands, which has also become an important research direction.^[^ ^138^, ^139^, ^140^ ^]^

#### 3.4.1 Polymer‐Derived Ceramic Composites

**Figure 10:**
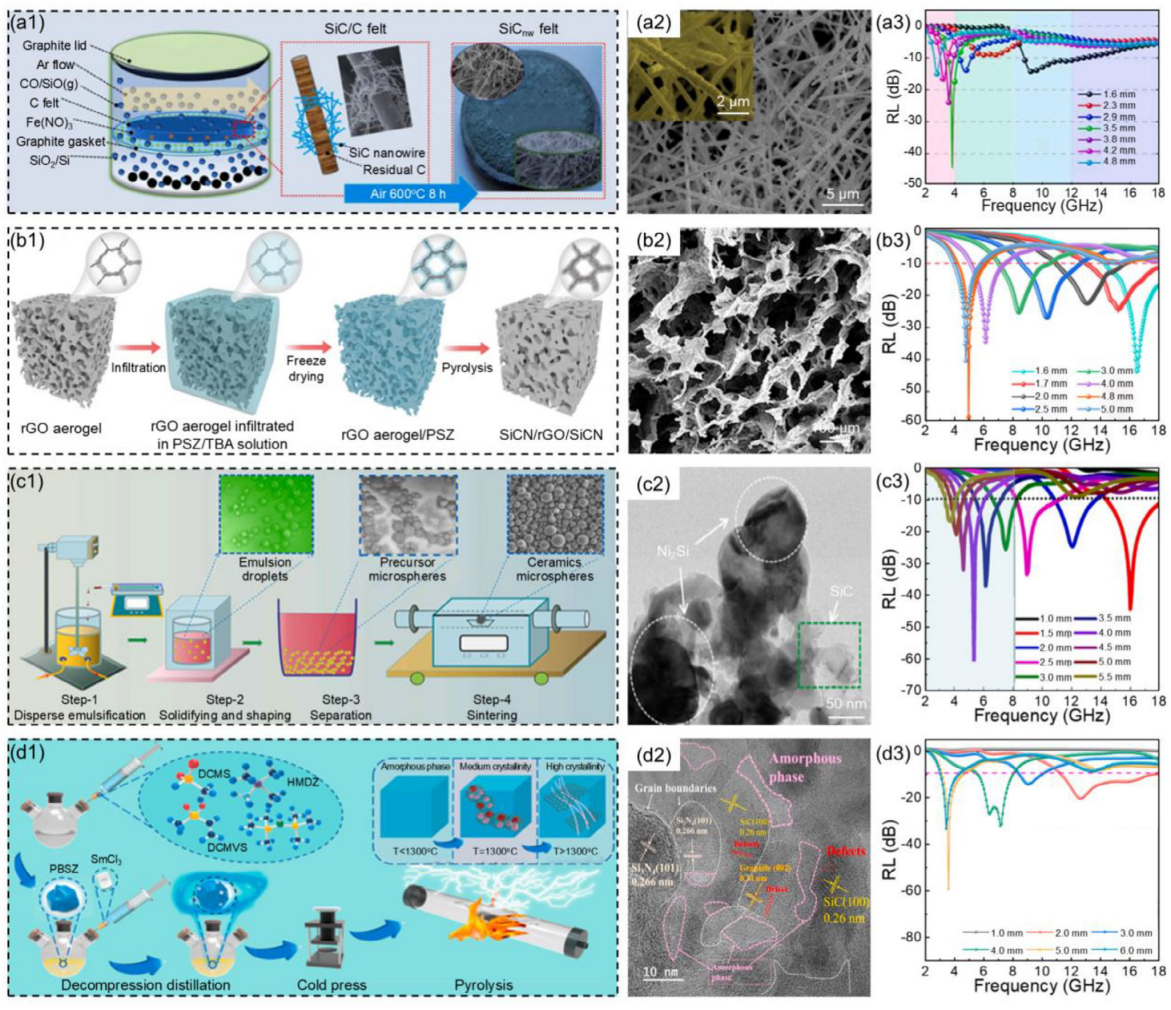
a1) Schematic illustration of the synthesis process of SiC nanowires felt, a2) SEM images, and a3) the reflection loss values at the corresponding thickness of bamboo‐shaped SiC nanowires felt.^[^ ^144^ ^]^ Copyright 2023, Elsevier, b1) Schematic illustration of the preparation process, b2) SEM image b3) and reflection loss values at the corresponding thickness for SiCN/rGO/SiCN.^[^ ^145^ ^]^ Copyright 2023, ACS, c1) Schematic illustration of the fabrication process, c2) TEM image of SiOCNi, and c3) reflection loss values at the corresponding thicknesses for SiOCM ceramic microspheres.^[^ ^146^ ^]^ Copyright 2023, Elsevier, d1) Schematic illustration of the prepared process, d2) TEM image, and d3) reflection loss values at the corresponding thickness for SiBCN ceramic composites.^[^ ^147^ ^]^ Copyright 2024, Elsevier.

Polymer‐derived method refers to the preparation of ceramics by directly pyrolyzing pre‐cured organic polymer precursors. While retaining the advantages of ceramic materials such as high temperature resistance, corrosion resistance, and good mechanical properties, it also has the excellent processability of polymer materials.^[^ ^141^, ^142^, ^143^ ^]^ Zhang et al.^[^ ^144^ ^]^ employed the carbon thermal reduction method combined with the gas–liquid–solid growth mechanism, regulating the silicon vapor concentration to prepare nanoscale SiC with different microstructures (linear, bamboo‐like, and worm‐like), which spontaneously overlapped to form a 3D network, resulting in SiC nanofibers felt (**Figure** **10**a1,a2). The results showed that when the sintering temperature was 1500 °C and the thickness of the bamboo‐jointed SiC nanofibers felt was 3.5 mm, the *RL*_min_ was −44.3 dB and the low‐frequency EAB was 0.6 GHz (3.6–4.2 GHz, Figure 10a3). Due to the high porosity (99.3%) of bamboo‐liked SiC nanofibers felt, the incident microwaves were prone to multiple scattering, thereby prolonging the propagation path of the microwaves and enhancing the dissipation of the electromagnetic waves, while also simultaneously meeting the lightweight requirements (density as low as 0.022 g cm^3^). Shao et al.^[^ ^145^ ^]^ constructed a 3D SiCN ceramic‐coated graphene aerogel (SiCN/rGO/SiCN, Figure 10b1,b2) using a precursor infiltration and pyrolysis process to create a conformal heterojunction interface. The results showed that when the precursor concentration was 100 mg mL^−1^, the pyrolysis temperature was 1200 °C, and the thickness of the SiCN/rGO/SiCN composites was 4.8 mm, the *RL*_min_ of the composites was −57.9 dB and the low‐frequency EAB was 1.9 GHz (4.1–6 GHz, Figure 10b3). This was primarily attributed to the abundant conformal heterogeneous interfaces in the 2D layered SiCN/rGO/SiCN honeycomb walls and the multiple scattering that was prone to occur in the 3D rGO porous framework, which enhanced the interface polarization capability and facilitated the dissipation of microwaves. Additionally, the conformal structure induced a unique pleated morphology of graphene nanosheets, thereby limiting the transmission of electrons and achieving impedance matching.

Wen et al.^[^ ^146^ ^]^ prepared novel intermetallic compound M_x_Si‐modified SiOC (SiOCM, M represented Fe, Co, and Ni) ceramic microspheres using the ceramic precursor emulsion method (Figure 10c1,c2). With the increase of pyrolysis temperature, SiOCM microspheres in situ generated M_x_Si, SiC, and graphitized carbon nanocrystals. The complex interface structure effectively enhanced the interface polarization effect and promoted the microwaves. When M was Ni and the thickness of the SiOCNi composites was 4 mm, the *RL*_min_ was −60.7 dB and the low‐frequency EAB was 1.4 GHz (4.7–6.1 GHz, Figure 10c3). Li et al.^[^ ^147^ ^]^ prepared SiBCN ceramics with adjustable crystallinity and defect nanoparticles by adding SmCl_3_ as the catalyst and combining with customized heat treatment temperatures (Figure 10d1,d2). The controllable crystallinity ensured good impedance matching, while the defective nanocrystals provided high polarization loss. When the mass fraction of SmCl_3_ was 1 wt.%, the heat treatment temperature was heated to 1000 °C for 1 h, then severally heated to 1200, 1300, and 1500 °C for 2 h. When the thickness of the SiBCN ceramic composites was 5.0 mm, the RL_min_ was −59.1 dB and the low‐frequency EAB was 1.7 GHz (3.1–4.8 GHz, Figure 10d3).

#### 3.4.2 Metamaterials

**Figure 11:**
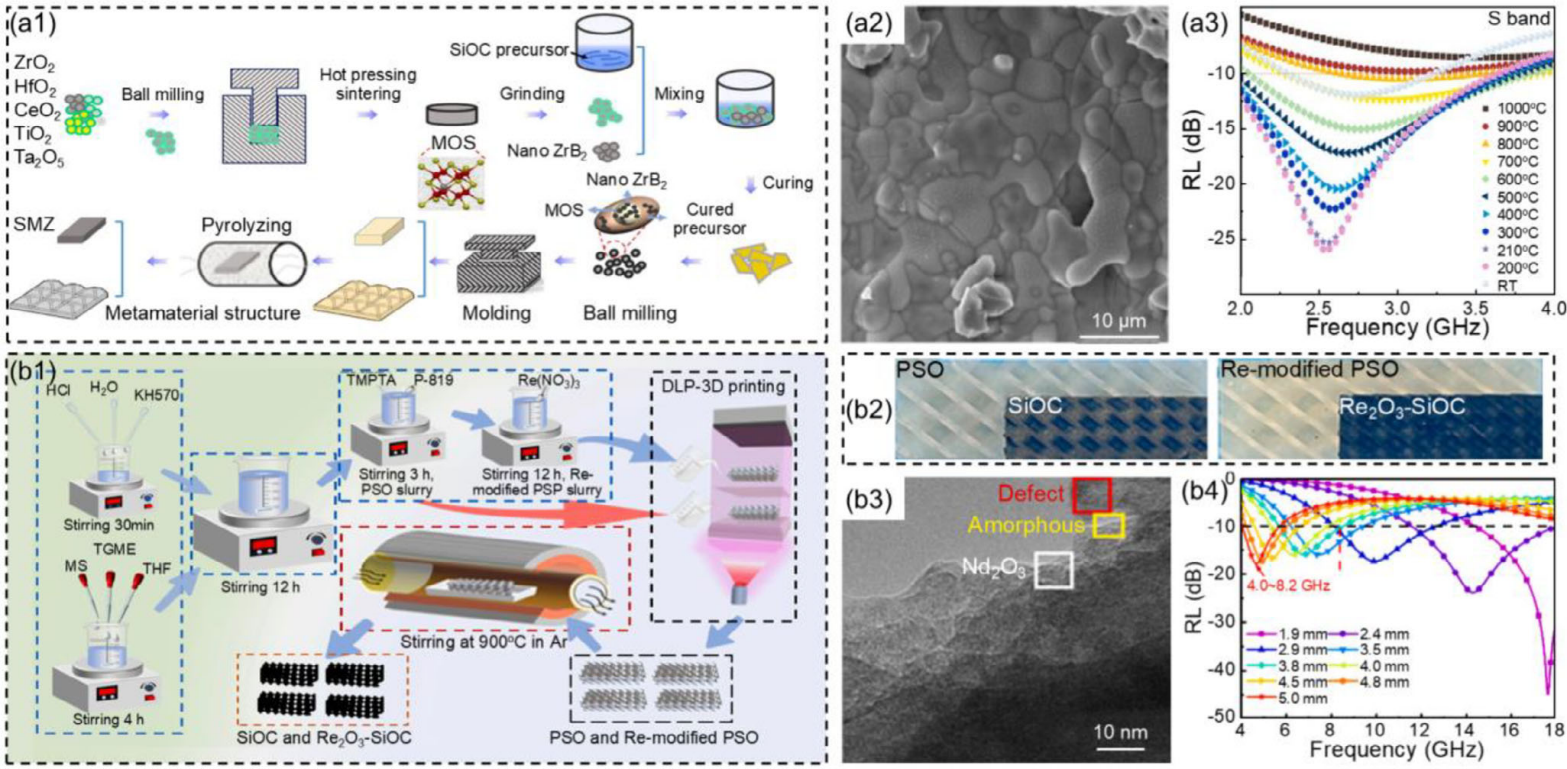
a1) Schematic illustration of the preparation process and a2) SEM image for the ceramic composites, a3) the reflection loss values of the metamaterial in the S band at different sintering temperatures.^[^ ^154^ ^]^ Copyright 2024, Elsevier, b1) Schematic illustration of the fabrication process, b2) digital photos, b3) TEM image, and b4) reflection loss values at corresponding thicknesses of Nd_2_O_3_‐SiOC metamaterials.^[^ ^155^ ^]^ Copyright 2025, Elsevier.

Ceramics can not only be directly used as MAMs but also be further designed as metamaterials.^[^ ^148^ ^]^ Metamaterials, as a new type of MAMs, possess extraordinary physical properties that natural materials do not have. Metamaterials can absorb most of the microwaves incident onto their surfaces without almost reflection or transmission.^[^ ^149^, ^150^ ^]^ Unlike traditional MAMs, metamaterials achieve high‐loss absorption of electromagnetic waves by triggering intense local resonance through their precisely designed resonant structures.^[^ ^151^, ^152^, ^153^ ^]^ Additionally, the structural unit design of metamaterials offers extremely high design flexibility. By adjusting the geometric parameters, arrangement patterns, and interlayer combinations of the unit structures, it is possible to precisely design the position, bandwidth, and intensity of absorption peaks, or even achieve tunable, wideband, or multi‐band low‐frequency absorption. This high degree of customization is unattainable with traditional materials. Li et al.^[^ ^154^ ^]^ prepared metal oxide semiconductor (MOS) by high‐temperature sintering process, and then dispersed MOS and nano‐zirconium boride (ZrB_2_) in polycarbosiloxane for cross‐linking and curing to obtain (MOS/ZrB_2_/SiOC) ceramic precursors. Subsequently, ceramic composites were prepared through high‐temperature pyrolysis at 1000 °C (**Figure** **11**a1,a2). The ceramic composites were further fabricated into metamaterials in the shape of a truncated quadrilateral pyramid. The results showed that the metamaterials exhibited excellent low‐frequency microwave absorption performance within the range of room temperature to 700 °C, almost covering the entire S band (Figure 11a3). Benefits from the addition of MOS and ZrB_2_, the permittivity of the composites could be adjusted without reducing the conduction loss of the composites, thereby improving the impedance matching of the metamaterials and broadening the high‐temperature absorption bandwidth.

Wang et al.^[^ ^155^ ^]^ were inspired by the Gyroid structure and used photopolymerization 3D printing as well as polymer‐derived ceramics technology to prepare metamaterials modified with rare earth oxides (Re_2_O_3_‐SiOC) that had triperiodic minimal surface (TPMS) metamaterials characteristics, where Re referred to holmium (Ho), neodymium (Nd), and yttrium (Y) (Figure 11b1–b3). The CST simulation showed that the Nd_2_O_3_‐SiOC metamaterials could cover the entire C band, and the effect was particularly significant within the thickness range of 2.9–5.0 mm (Figure 11b4). This was mainly due to the excellent impedance matching achieved by its 3D array design and unique porous structure. Furthermore, the complex stepped structure led to multiple scattering of microwaves, thereby increasing the loss and concentrating the power dissipation at the pores and shells of the 3D TPMS metamaterials.

### 3.5 Multiphase Hybrid Composites

Multiphase hybrid microwave absorption composites usually refer to materials that contain multiple different phases, such as combinations of metals, ceramics, and polymer components, with different components tightly bonded through chemical bonds, interface coupling, or microstructural design.^[^ ^26^, ^156^ ^]^ As an emerging 2D transition metal MXene, it demonstrates unique intrinsic advantages in the field of microwave absorption due to high electrical conductivity, abundant surface functional groups, tunable dielectric properties, and layered structure.^[^ ^157^, ^158^ ^]^ These characteristics make MXene an ideal unit for constructing multi‐phase hybrid composite materials, enabling the activation of interfacial polarization mechanisms between MXene and other materials through interfacial design.^[^ ^159^, ^160^, ^161^ ^]^ Additionally, the dimensional differences of multiphase materials can be utilized to design other composites. At the same time, by combining the interaction mechanisms among different components, multiple losses can be coordinated, thereby breaking through the development goals of thin thickness, wide frequency band, low density, and strong absorbing of low‐frequency MAMs.^[^ ^162^ ^]^

#### 3.5.1 MXene Composites

**Figure 12:**
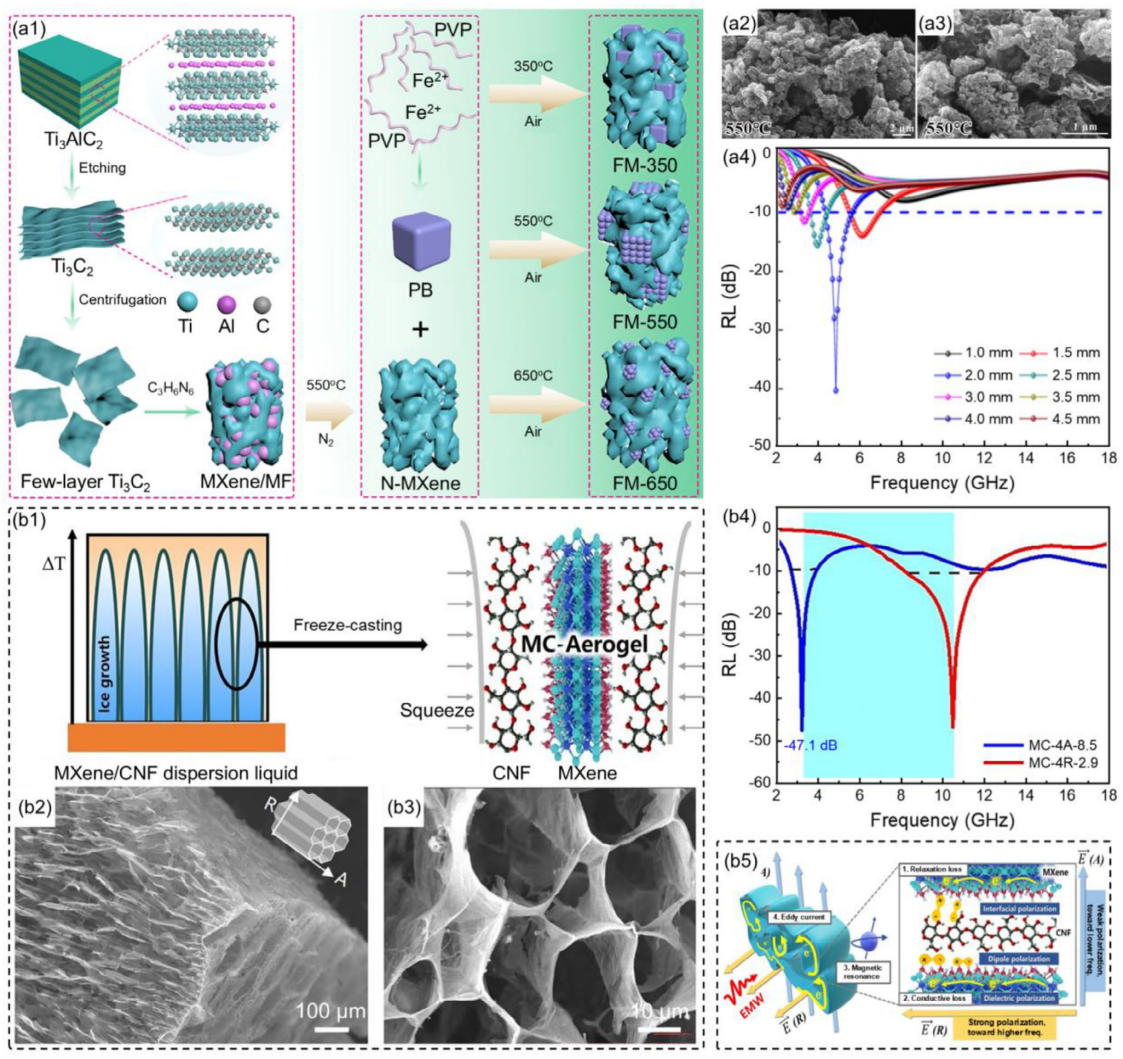
a1) Schematic illustration of the fabrication process, a2,a3) SEM images, and a4) reflection loss values at corresponding thicknesses for Fe/MXene composites.^[^ ^164^ ^]^ Copyright 2023, ACS, b1) Schematic illustration of the preparation process, b2,b3) SEM images, and b4) reflection loss values at corresponding thicknesses for MXene/CNF aerogel composites, b5) absorption mechanisms of MXene/CNF aerogel composites.^[^ ^165^ ^]^ Copyright 2024, Springer Nature.

The high electrical conductivity of MXene is a double‐edged sword. While it exhibits strong dielectric loss and polarization loss, it also causes the problem of poor impedance matching.^[^ ^163^ ^]^ To enhance the impedance matching and microwave attenuation capacity of materials, MXene is usually combined with different materials to obtain high‐performance multiphase hybrid microwave absorption composites that integrate functions such as microwave absorption, conductivity, and voltage resistance.^[^ ^45^ ^]^ Liu et al.^[^ ^164^ ^]^ prepared 3D honeycomb MXene using the template method and further employed the thermal annealing process to anchor Prussian blue microcubes and their derivatives onto the MXene surface to prepare Fe/MXene composites (**Figure** **12**a1–a3). Utilizing the advantages of a 3D honeycomb structure and magnetic‐dielectric synergy, the Fe/MXene composites exhibited excellent microwave absorption performance. When the hot annealing temperature was 550 °C and the thickness of the Fe/MXene composites was 2 mm, the *RL*_min_ of the composites was −40.3 dB and the low‐frequency EAB was 1.4 GHz (4.2–5.6 GHz, Figure 12a4). The simulation indicated that the RCS attenuation value was all lower than −15 dB m^2^ when the incident angle was 0° to 180°, demonstrating the excellent radar wave attenuation capability of Fe/MXene composites. Furthermore, Fe/MXene composites also possessed excellent flexibility and thermal insulation performance.

Huang et al.^[^ ^165^ ^]^ combined fluorine‐doped MXene, which exhibited high microwave responsivity, with the dielectric material cellulose nanofibers (CNF) to construct highly anisotropic electromagnetic response cavity MXene/CNF aerogel composites (Figure 12b1–b3). By controlling the dipole polarization and resonance response characteristics of the MXene/CNF aerogel composites, the low‐frequency absorption efficiency of the 3D cavity was enhanced. This controlled polarization mechanism could cause the microwave absorption interval to shift significantly from the high‐frequency X band to the low‐frequency S band. When the mass fraction of MXene was 40 wt.%, the opening direction of the aerogel was perpendicular to the microwave propagation direction, and the thickness of the MXene/CNF aerogel composites was 2.9 mm, the composites showed the *RL*_min_ of −47.1 dB and the EAB of 3.8 GHz (8.2–12.0 GHz, Figure 12b4). When the opening direction of the aerogel was parallel to the microwave propagation direction, and the thickness of the MXene/CNF aerogel composites was 8.5 mm, the composites exhibited the *RL*_min_ of −47.9 dB and the EAB of 1.1 GHz (2.8–3.9 GHz, Figure 12b4). The deep mechanism behind the shift of the absorption frequency band from high frequency to low frequency was attributed to the electromagnetic coupling generated by structure‐induced orientation polarization and derived magnetic resonance (Figure 12b5).

#### 3.5.2 Other Composites

Designing other composites by using the different sizes and shapes of multiphase materials can effectively adjust electromagnetic parameters, enhance interface effects, improve impedance matching, and thereby enhance microwave absorption performance.^[^ ^166^, ^167^ ^]^ Through bionic structural design, composites can be endowed with rich pores and interfaces, thereby enhancing the impedance matching and dielectric loss of the materials.^[^ ^110^, ^168^, ^169^ ^]^

**Figure 13:**
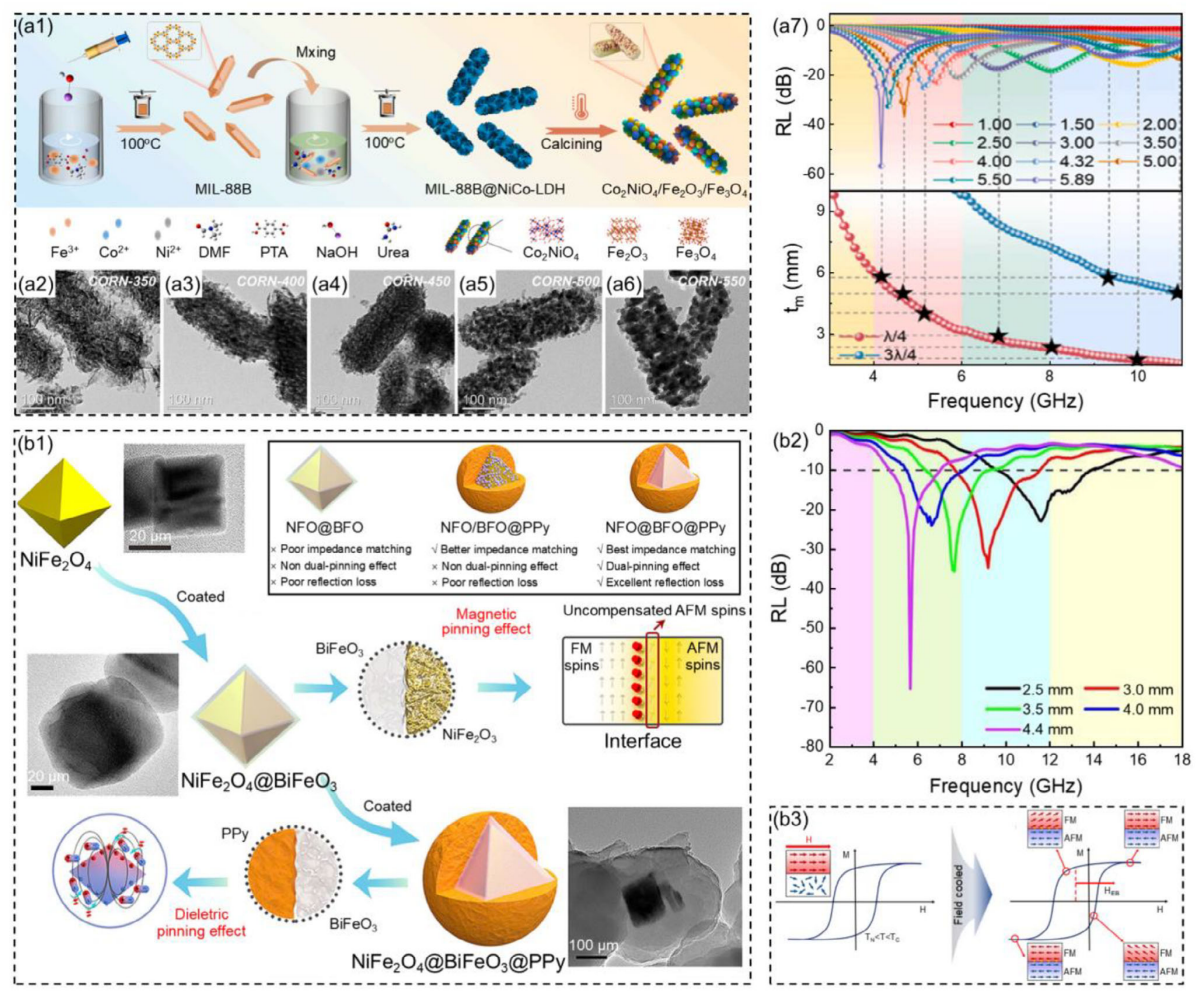
a1) Schematic illustration of the preparation process and a2–a6) TEM images for the Co_2_NiO_4_/Fe_2_O_3_/Fe_3_O_4_ ternary nanocomposites, a7) the reflection loss values and matching thicknesses of the Co_2_NiO_4_/Fe_2_O_3_/Fe_3_O_4_ composites at the corresponding thickness on the thermal reduction temperature of 400 °C.^[^ ^170^ ^]^ Copyright 2024, Elsevier, b1) Schematic illustration of the fabrication process, b2) reflection loss values at corresponding thicknesses and b3) pinning mechanism for NFO@BFO@PPy composites.^[^ ^2^ ^]^ Copyright 2024, Springer Nature.

Yu et al.^[^ ^170^ ^]^ designed and fabricated ternary nanocomposites of Co_2_NiO_4_/Fe_2_O_3_/Fe_3_O_4_, simulating the bionic corn‐structure (**Figure** **13**a1–a6). The Co_2_NiO_4_/Fe_2_O_3_/Fe_3_O_4_ composites featured abundant heterogeneous interfaces, a large specific surface area, and pores. These features effectively enhanced interface polarization, optimized impedance matching, and also induced low‐frequency natural resonance. The heterogeneous interface engineering of bionic corn‐structure ternary nanocomposites enhanced the synergy effect of dielectric loss and magnetic loss in the low‐frequency band. When the thermal reduction temperature was 400 °C, the *RL*_min_ of the Co_2_NiO_4_/Fe_2_O_3_/Fe_3_O_4_ composites at 4.4 GHz was −55.2 dB (Figure 13a7). Core–shell structure (magnetic core and dielectric shell) composites not only utilized the advantages of core and shell materials, but also induced interface polarization phenomena at the heterogeneous interfaces. The polarization phenomenon enhanced the dielectric loss of materials and improved the absorption capacity for microwaves. Wang et al.^[^ ^2^ ^]^ used a wet chemical method to construct a double‐layer core–shell structure NiFe_2_O_4_@BiFeO_3_@Polypyrrole (NFO@BFO@PPy, Figure 13b1). When the thickness of the NFO@BFO@PPy composites was 4.4 mm, the *RL*_min_ of the composites was −65.3 dB and the low‐frequency EAB was 2.3 GHz (4.7–7.0 GHz), almost covering the C band (Figure 13b2). The fundamental reason was the introduction of a double pinning mechanism in the NFO@BFO@PPy composites, which established the magnetic‐electric bias interface (Figure 13b3). This non‐uniform interface induced magnetic pinning of magnetic moments in ferromagnetic NFO and dielectric pinning of dipole rotations in PPy, enhancing impedance matching and attenuation capabilities in the low‐frequency band.

## 4 Conclusions and Perspectives

The basic principle of microwave absorption and the influencing factors of low‐frequency microwave absorption performance are briefly introduced. The research progress for carbon‐based composites, magnetic‐based composites, polymer‐based composites, ceramic‐based composites, and multiphase hybrid composites is systematically reviewed. And the intrinsic relationship of micro‐structure as well as macro‐performance and the microwave absorption mechanisms of different composites are deeply explored. Certain progress has been made in the controllable preparation and structural design of low‐frequency MAMs, but there are still the following key scientific and technological bottlenecks that need to be solved urgently.

1. Carbon‐based materials (such as carbon fiber, graphene, and carbon nanotubes) demonstrate significant advantages in terms of lightweight and broadband microwave absorption, but they still face core challenges such as poor interface compatibility, poor structural stability, and complex manufacturing processes. These challenges can be addressed by constructing core–shell structure of carbon‐coated magnetic particles to synergize dielectric/magnetic loss, and by doping carbon materials with elements such as nitrogen and sulfur to regulate their electronic structure and enhance low‐frequency polarization effects. This approach can accelerate the practical application of carbon‐based materials in low‐frequency fields such as 5G communications and stealth technologies.
2. Binary magnetic alloys (FeNi, CoNi, and FeCo) can absorb microwaves through inherent magnetic loss, balancing the low‐frequency impedance matching and enhancing the low‐frequency microwave absorption performance. However, when binary magnetic alloys are compounded with other materials, the significant difference in density could lead to filler stratification. The traditional limitations can be overcome through core–shell structure design and anisotropy optimization combined with external field‐induced processes such as magnetic and electric fields. Furthermore, in situ surface oxidation techniques are employed to regulate interfaces and dispersion, effectively balancing the density differences between magnetic materials and composites. And theoretically explore a new mechanism to break through the Snoek limit and establish a low‐frequency microwave absorption model of nanomaterials.
3. Polymer‐based (polyimide, polytetrafluoroethylene, polydimethylsiloxane) microwave absorption composites offer the advantages of excellent design flexibility and tunable electromagnetic parameters. To meet the diverse demands of different application fields, the development of multifunctional integrated materials and optical/magnetic/thermal smart responsive materials, as well as polymer‐based composites that support dynamic performance regulation, has become the future development trend.
4. Ceramic‐based composites show significant application potential in aerospace, 5G communications, and other fields, but still face the challenge of performance degradation at high temperatures. This can be achieved by forming self‐healing protective layers on the surface of high‐entropy oxides, and combined with gradient impedance design to optimize microwave energy dissipation pathways. These approaches synergistically resolve the integrated challenges of oxidation resistance, ablation resistance, and functional integration in high‐temperature environments.
5. Multiphase hybrid composites can combine dielectric loss (interfacial polarization and polarization relaxation), magnetic loss (eddy current loss and natural resonance), and multiple scattering mechanisms to achieve a dynamic balance between impedance matching and attenuation capacity. Furthermore, machine learning‐assisted calculations can be used to predict the multi‐component–structure–performance relationship through algorithms such as artificial neural networks, improving material design efficiency.

## Conflict of Interest

The authors declare no conflict of interest.
